# Pseudonatural Products for Chemical Biology and Drug Discovery

**DOI:** 10.1021/acs.jmedchem.5c00643

**Published:** 2025-07-01

**Authors:** Luca C. Greiner, Axel Pahl, A. Lina Heinzke, Barbara Zdrazil, Andrew R. Leach, Robert J. Young, Paul D. Leeson, Herbert Waldmann

**Affiliations:** † 28268 Max-Planck Institute of Molecular Physiology, Department of Chemical Biology, Otto-Hahn-Strasse 11, Dortmund 44227, Germany; ‡ European Molecular Biology Laboratory, 265353 European Bioinformatics Institute (EMBL-EBI), Wellcome Genome Campus, Hinxton CB10 1SD, Cambridgeshire, U.K.; § Blue Burgundy Ltd., Bedfordshire, Ampthill MK45 2AD, U.K.; ∥ Paul Leeson Consulting Ltd., Nuneaton, Warwickshire CV13 6LZ, U.K.; ⊥ Technical University Dortmund, Faculty of Chemistry and Chemical Biology, Otto-Hahn-Strasse 6, Dortmund 44227, Germany

## Abstract

Natural product (NP) structures have provided invaluable inspiration for the discovery of bioactive compound discovery. In the pseudonatural product (PNP) concept unprecedented combinations of NP fragments combine the biological relevance of NPs with exploration of wider chemical space by fragment-based design. We describe the principles underlying the PNP design and discovery of selected PNPs with unexpected or novel bioactivity. Cheminformatic analyses of ChEMBL 32, the Enamine screening library, phase 1–3 clinical compounds, and approved drugs reveal that ca. 1/3 of historically developed biologically active compounds and of currently commercially available screening compounds are PNPs, and that PNPs are increasing over time. PNPs are 54% more likely to be found in clinical compounds versus nonclinical compounds, and 67% of recent clinical compounds are PNPs. 63% of the core scaffolds in recent clinical compounds are made up of just 176 NP fragments, which suggests that PNPs open up a multitude of unexploited opportunities for drug discovery.

## Graphical abstract

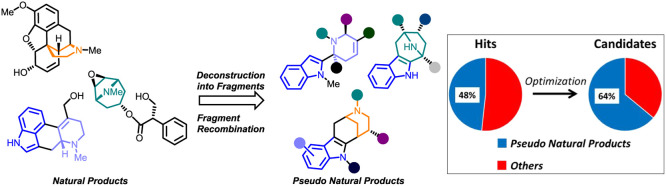

## 1 Introduction

Bioactive small molecules have been widely applied as chemical probes for the investigation of complex cellular mechanisms,^1,2^ and they represent the prevalent chemical modality among marketed drugs.^3,4^ Hence, novel principles for the design and discovery of small molecules that populate the biologically relevant chemical space are of high importance and in high demand for both chemical biology and medicinal chemistry research.

Biological relevance will be assured if the design concepts for new chemical entities draw from compound classes that encode binding to protein targets in their three-dimensional structure and, therefore, have proven to be meaningful to nature. In evolution, nature has explored biologically relevant chemical space through natural products (NPs), and these biologically prevalidated small molecules have been a rich source of therapeutics as well as an inspiration for molecular design principles.^5^ Indeed, processes of natural selection are likely influenced by the ability of proteins to interact with or transport beneficial dietary molecules to the benefit of the organisms encoding particular sites through genetic variation.^6^ However, because of evolutionary constraints like structural conservatism in NPs and proteins and the fact that evolution is slow, NPs occupy only a limited fraction of NP-like chemical space.^7–8^ In response to this limitation, several molecular design principles have been introduced to motivate the synthesis of natural product-inspired compound collections and their exploration in chemical biology and medicinal chemistry research.^9–11^

In diversity-oriented synthesis (DOS), structurally diverse small molecule collections are synthesized by employing a three-phase synthesis strategy consisting of a build, a couple, and a pair phase.^9^ Varying the combination of the building blocks and group pairs ensures stereochemical and skeletal diversity, high sp^3^ content, richness in stereogenic centers, and varying scaffold combinations, and due to these properties, the resulting compound collections have been considered NP-inspired. DOS has recently been applied in tandem with the DNA-encoded libraries (DEL) approach to generate relatively large libraries.^10^ Although DOS allows fast exploration of a large and diverse chemical space and has yielded biologically valuable compounds, most of the investigated chemical space is not necessarily biologically relevant.

NP-like compound collections rich in skeletal diversity can efficiently be generated through the complexity-to-diversity (CtD) approach.^11^ In CtD, readily accessible NPs are subjected to skeletal distortions such as ring cleavage, ring expansion, and intramolecular rearrangements to yield new compounds with novel scaffolds that retain NP-like features. CtD is an effective strategy for exploring the chemical space surrounding given NPs. However, this concept is limited by the restricted number of NPs readily available at scale. Biology oriented synthesis (BIOS) employs the evolutionary logic that proteins and NPs have coevolved to encompass only a small fraction of chemical space, such that the core scaffolds of NPs and the corresponding binding pockets of proteins are conserved. Using this logic, complex NPs are computationally simplified to truncated parent scaffolds via successive removal of rings, functional groups, and appendages (Figure 1).^8^ The resulting scaffolds are synthetically more tractable than their corresponding NPs, yet they retain biologically relevant characteristics. However, BIOS is limited both biologically and chemically, because the core scaffolds represent only the chemical and biological space explored by nature through biosynthesis.

**Figure 1:**
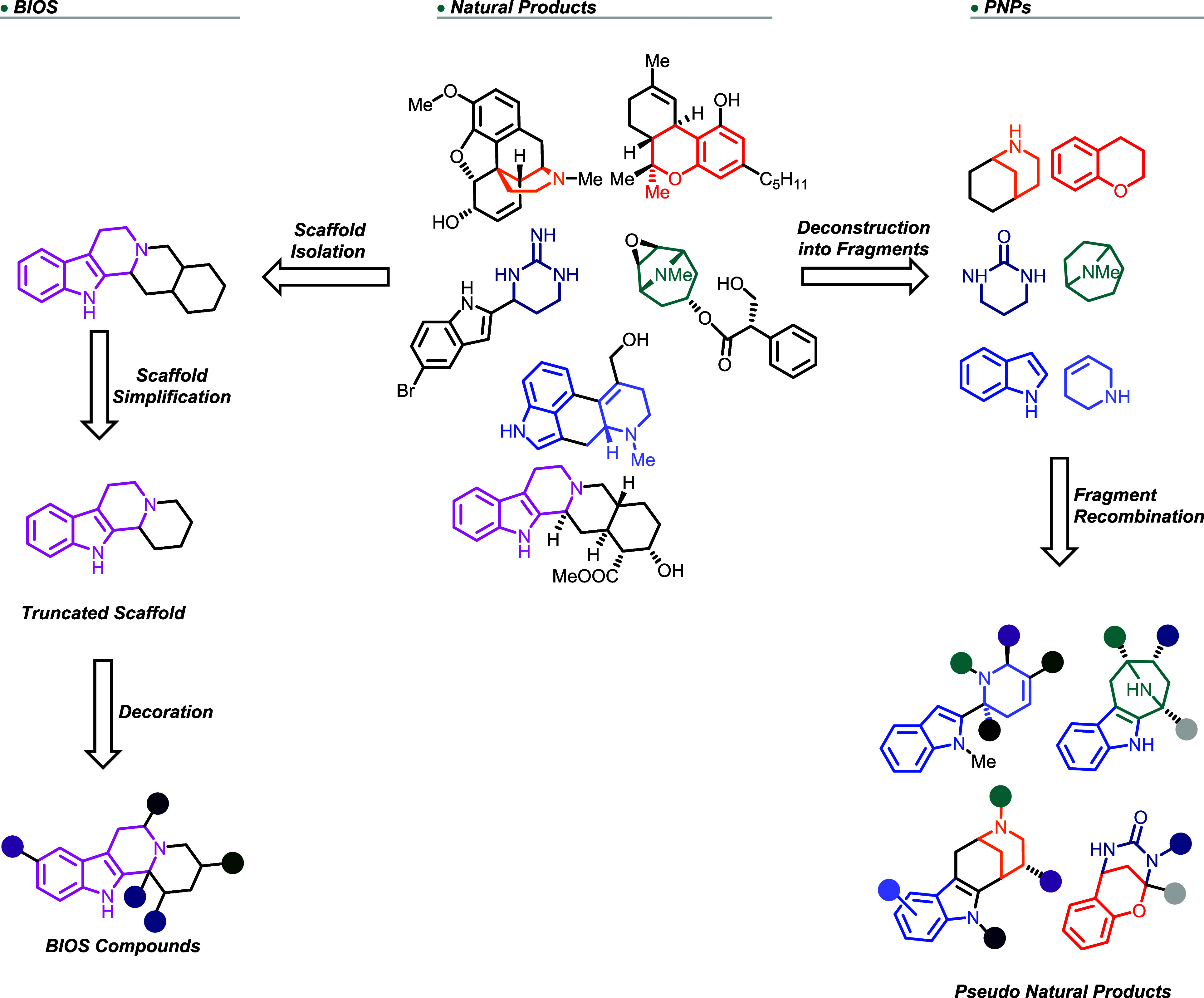
Principles of biologically oriented synthesis (BIOS) and PNP design.^8,16^

The design principles discussed above have successfully been applied to generate compound collections enriched with biologically active members, but each is limited in the exploration of biologically relevant chemical space. To overcome these limitations while still preserving the biological relevance of NPs, the pseudonatural product (PNP) concept was developed.^12,13–18^ The PNP principle combines natural product structures, and, thereby, the biological prevalidation of NPs with fragment-based design.^19–21^ Natural products can be considered combinations of fragments or can be fragment-sized, and NP fragments can be isolated *in silico* by computational deconstruction (Figure 1).^20^ In PNP design and synthesis, NP fragments or fragment-sized NPs are recombined *de novo,* unconstrained by evolutionary pathways, and in arrangements not found in nature. Thereby the PNP principle enables the rapid exploration of an untapped biologically relevant chemical space. NP fragments are combined to scaffolds with differing NP-fragment combinations^22^ and/or differing fragment orientations,^12^ that are inaccessible through known biosynthetic transformations while retaining the biological relevance of NPs.

PNP design conceptually differs from the synthetic NP-hybridization strategy. In NP-hybridization, NPs are combined to interact with the biological targets of their parent NPs.^23^ However, the novel arrangements of NP fragments in PNPs are expected to yield compounds with novel biological activities and targets not related to their guiding NPs.

In this Perspective, we discuss and focus on the design principles guiding the subsequent synthesis of PNP libraries and their biological assessment. For in-depth discussion of the evolutionary relationships underlying the PNP principle, the reader is referred to previous reviews.^16,22^ We briefly explain that PNP design can be regarded as an accelerated chemical equivalent to the evolution of the NP structure in nature. We show that, unexpectedly, numerous PNPs were unintentionally synthesized historically such that they represent a significant segment of the currently known bioactive compounds. Our analysis further demonstrates that a large number of PNPs is readily available from commercial sources, i.e., without the need to develop challenging and asymmetric complexity-generating reaction sequences. Finally, we extend our analysis to compounds investigated in clinical phases I–III and marketed drugs and provide evidence that transition from discovery through to clinical phases and the market is significantly higher for PNPs as compared to non-PNPs across the majority of the major target classes.

## 2 Pseudonatural Product Design Principles

### 2.1 Identification of NP-Fragments

NP fragments for PNP design were identified through analysis and fragmentation of NP structures by means of an algorithm which resembles the structure simplification-logic previously developed in the establishment of the BIOS principle.^20^ In a first step, the side chains of the 226,000 NPs stored in the Dictionary of Natural Products were pruned, and scaffold structures were reduced successively one ring at a time. Atom hybridization and stereogenicity were stored. Successive ring system deconstruction yielded 751,577 NP fragments. Subsequent filtering according to relaxed “rule of three” criteria, i.e., Alog *p* < 3.5, molecular weight 120–350 Da, ≤3 hydrogen-bond donors, ≤6 hydrogen-bond acceptors, and ≤6 rotatable bonds yielded 160,000 fragments.^24^ Clustering of these fragments according to Tanimoto fingerprint similarity resulted in 2000 natural-product fragment clusters. Within the clusters, structural similarity is high; between the clusters, similarity is low. Notably, analysis for number of N- and O-atoms and H-bonds and richness in sp^3^-configured centers revealed that the properties of the cluster members represent characteristic properties of the guiding natural products and of the NP fragments. Thus, collectively, the identified fragments share key structural parameters of the guiding NPs, and thereby, their biological relevance encoded in the structure. Hence, *de novo* fragment recombination should yield structurally novel and still biologically relevant NP-inspired pseudonatural products.

### 2.2 PNP Design-Principles and Strategies

In PNP design, the initial steps are choice of the fragments and their combination. For these steps, general considerations are

1) Fragments preferably should embody stereogenic centers, and/or in their combination new stereogenic centers should be created. This reasoning takes into consideration that stereogenic character is beneficial for producing selective biological activity and chiral compounds demonstrably improve transition rates from discovery through development stages.^19^ It also suggests that for PNP synthesis, complexity-generating asymmetric transformations should be applied or may even have to be developed. In such syntheses, individual fragments may be formed (e.g., by means of a cycloaddition), instead of simply linking preformed fragments.

(2) Fragments should contain complementary heteroatoms, i.e., rich in nitrogen (more frequently found in drugs than in NPs) and oxygen (more frequent in NPs than in drugs) to enable a variety of precedented interactions with biological targets.

(3) Fragments of NPs with diverse bioactivities should be combined to increase the biological relevance of the resulting PNPs.

(4) Biosynthetically unrelated fragments should be combined to maximize the structural uniqueness of the PNP scaffold and to encode interactions with different proteins during biosynthesis. However, we note that this design principle may be of only secondary relevance. In a recent PNP synthesis, biosynthetically related fragments were combined in different arrangements which yielded different bioactivity.^25^ This finding suggested that different combination types may be of greater relevance than different biosynthetic origin. Biosynthetic considerations can also be taken into account in the choice of chemistry employed for fragment linking. For instance, indofulvin PNPs were obtained by linking fragments employing a transformation that is not in the current biosynthetic repertoire, i.e., an oxa-Pictet–Spengler reaction.^26^

NP fragment combinations can broadly be divided into connectivity patterns in which the combined fragments share common atoms (Figure 2, panel a, **1–9**), or in which they are connected through intervening atoms (Figure 2, panel b, **10–18**), and these connectivity types may actually occur in combination (Figure 2, panel c, **19–24**).

**Figure 2:**
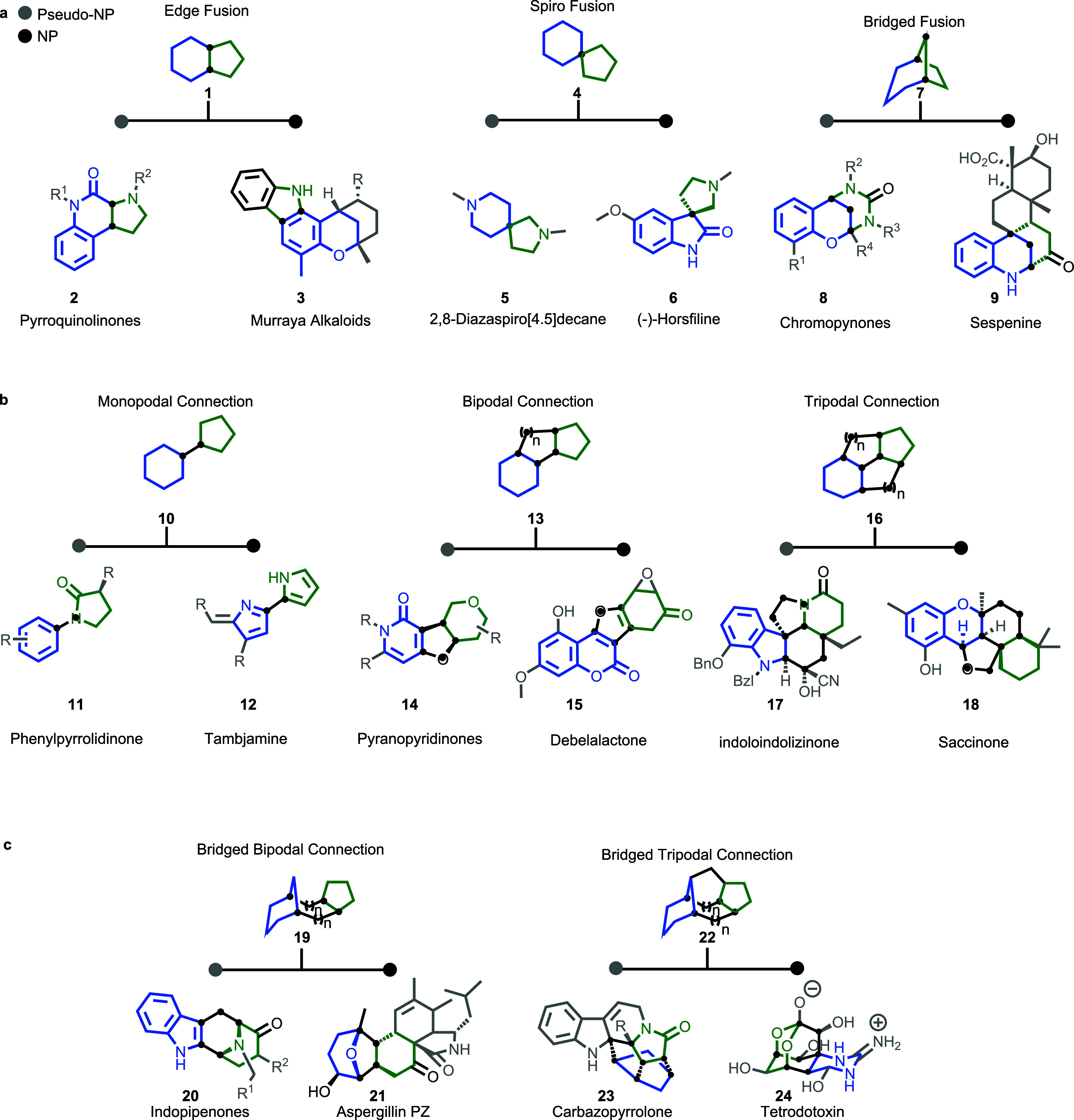
PNP scaffold design principles: **a)** NP fragments can be combined in various connectivity patterns, utilizing a fusion edge, spiro, or bridge with shared atoms. **b)** Combinations of NP fragments can involve different connection types with intervening atoms, including mono-, bi-, or tripodal connections. **c)** Fragment combinations can also include both connectivity patterns, such as bridged bipodal or bridge tripodal connections.

Fragment combinations through shared atoms may feature different connectivity patterns, i.e., fusion edge **1** as in pyrroquinoline PNPs **2** and in Murraya alkaloids **3**.^27,28^ The fusion spiro connection **4** is illustrated by 2,8-diazospiro[4.5]­decane **5** and the NP (-)-horsfiline **6**, and fusion bridge **7** is represented by chromopynone PNPs **8** and the NP sespenine **9**.^29,30^

Fragment combination by means of intervening atoms may be monopodal **10** as for instance in phenylpyrrolidone **11** and the NP tambjamine **12**.^31^ A bipodal connection **13** is found in pyrano-pyridinone PNPs **14** and debelactone **15**,^32^ and a tripodal connection **16** characterizes structure **17** and the NP saccinone **18**. A fragment combination including both general connectivities would for instance be bridged bipodal **19** as shown by indopipenones **20** ^33^ and aspergillin PZ **21**, or bridged tripodal **22** as for instance in carbazopyrrolone **23** and tetrotodoxin **24**.

In the design and synthesis of PNP collections, the same or different fragments can be combined *de novo* in different connectivity patterns to maximize structural novelty and chemical and biological diversity. For instance, combination of four fragment-sized natural products with chromanone- and indole fragments in different connectivities, stereo- and regioisomeric arrangements yielded a chemically and biologically diverse compound collection (see Section 4).^25,34^

Alternatively, instead of different fragment connections (Figure 3, compare **25** and **26**) fragment connectivity may be kept, but different connection points may be employed, i.e., through different regioisomeric arrangements as shown for pyrroquinolinones **26**, **27,** and **28**.

**Figure 3:**
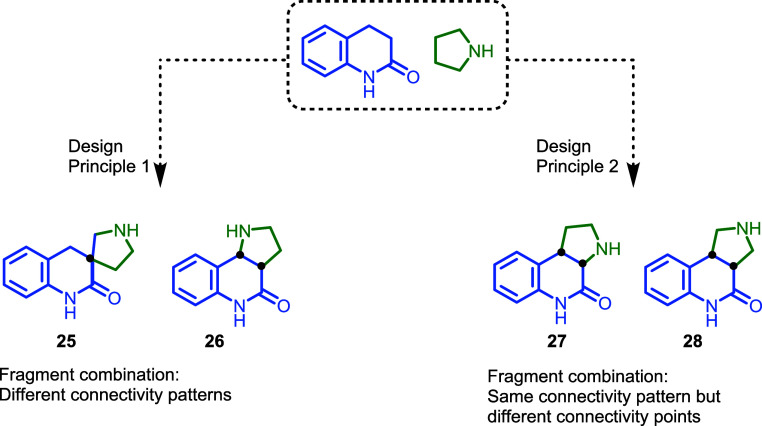
General PNP design principles. Pseudo NPs are generated via a combination of different connectivity patterns, varying connectivity points while maintaining the connectivity pattern, or through the combination of more than two fragments.

The number of fragments to be preferably combined in PNPs deserves particular attention. As discussed below, analysis of the ChEMBL database^35^ revealed numerous PNPs for which bioactivity was reported.^36^ In the overwhelming majority (95.6%) of these bioactive PNPs, two, three, or four NP-fragments are combined in monopodal, edge, fusion bridge, fusion spiro, or bipodal edge connectivity. In addition, cheminformatic analysis of PNPs in clinical phases and in marketed drugs revealed that they contain on average two more NP fragments than reference compounds.^37^ These findings suggest that in PNP collection design and synthesis, preferably more than two fragments should be considered for combination in a given PNP class. The preferred number of fragments combined may for instance reflect the volume of small molecule binding sites on and in proteins.^38^

In addition to the number of combined fragments, fragment size and structure also deserve attention. The computational analysis of NPs to identify NP-fragments applies the “relaxed” rule of three (see above) and leads to fragments of different size and structural complexity, which also include small carbocycles and heterocycles. These smaller fragments often resemble building blocks employed widely in drug discovery in general and seemingly without a particular connection to NP structure.

However, it should be noted that structure, size, and complexity of NPs may differ widely and that, in fact, NPs themselves may be fairly small and already fragment-sized, i.e., the NPs themselves may fulfill the “relaxed” fragment criteria defined above and thus be fragment-sized. For instance, the alkaloid coniine is a monosubstituted piperidine, kainic acid is a structurally simple pyrrolidine derivative, and salicylic acid, the core scaffold of aspirin, the most successful drug ever, is a simple disubstituted benzene. At first glance, none of them might seem particularly relevant to bioactive compound design and drug discovery. However, cheminformatic analysis of PNPs in bioactive compounds assembled in the ChEMBL database, in clinical phases, and in marketed drugs (see below) revealed that fragment combinations including such small NP-derived carbo- and heterocycles occur frequently in bioactive compounds and in drugs which can be considered PNPs. Therefore, the use of such small fragments in PNP design will yield new biologically relevant compound classes, and their purposeful inclusion in PNP design is of high potential value.

In addition to compound-focused considerations, synthetic matters must be taken into account. For monopodal connection of differently functionalized fragments, well established and widely applicable methods are readily available.^39^ In contrast, PNPs with fusion bridge- or fusion-spiro connectivity, which for instance can include multiple stereocenters or multiple NP fragments, are more challenging to synthesize and, thus, require more sophisticated methods and synthetic pathways.

We also note that the PNP principle can advantageously be combined with other approaches developed for the design of natural product-inspired compound classes. For instance, PNP design can be amalgamated with Hergenrother’s CtD approach. An example is the combination of pyrrolidine NP fragments derived from alkaloids such as nicotine **29** and kainic acid **30**, with a variety of fragment-sized α-methylene-sesquiterpene lactones like parthenolide **31**, santonine **32**, and micheliolide **33** (Figure 4, panel a).^11,40^ These lactones can be seen as products of NP ring distortion as proposed in CtD. Combination of both fragment classes results in new bioactive chemically and biologically diverse pseudosesquiterpenoid alkaloids.

**Figure 4:**
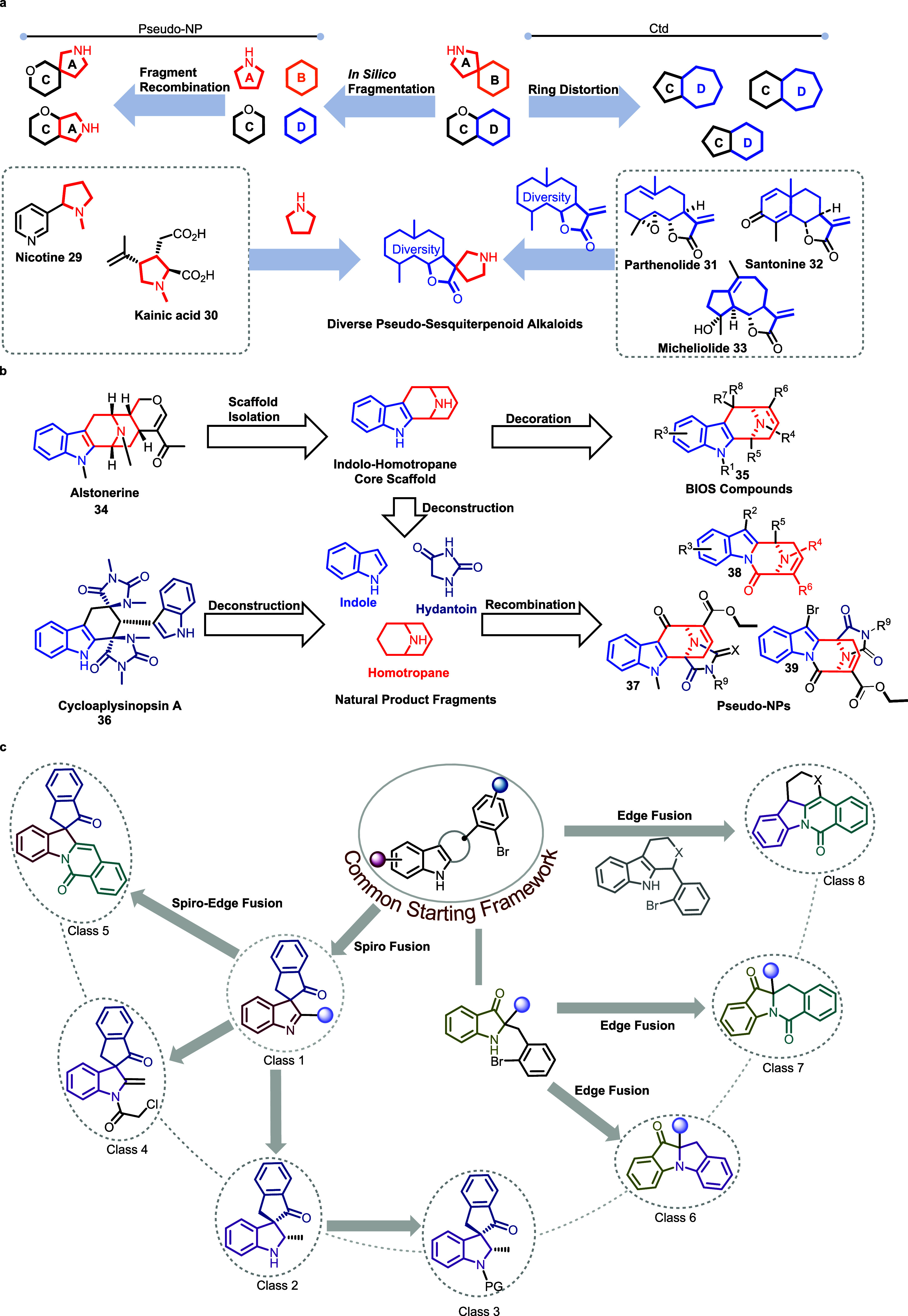
Combination of complementary design principles. **a)** Combination of the PNP and the CtD approaches yields diverse pseudosesquiterpenoid alkaloids. **b)** Combination of the PNP logic with the BIOS. **c)** Combination of DOS with the PNP logic giving diverse pseudonatural products (dPNPs). Structures **34**, **35**, **37**, **38,** and **39** as well as class 5 and class 8 contain an edge fusion of an aromatic and an aliphatic ring, and we consider them PNPs. (a–c) Adapted and modified from Liu et al., Aoyama et al., and Bag et al.,^40–42^ under CC-BY4.0 and CC-BY-NC-ND 4.0.

The indolo[3.3.1]­homotropane fragment is present in various compounds with diverse bioactivities and defines the core structure of macroline alkaloid Alstonerine **34**. Simplification of its structure to indolo-homotropane and decoration of the scaffold resulted in BIOS compounds **35**. However, deconstruction of the indolo-homotropane scaffold and combination of the fragments with fragments obtained from the sarpagine alkaloid cycloaplysinopsin **36** conceptually merge BIOS and the PNP principle. The novel PNP class **37**-**39** contains unprecedented tubulin modulators.^41^

Combination of the DOS concept,^9^ which can generate scaffold diversity by using the build-couple-pair strategy with available starting materials, and the PNP logic, which combines NP fragments in unprecedented arrangements, can give rapid access to a diverse PNP (dPNP) library. In the combined approach, a functionalizable common intermediate is generated, which is then transformed into diverse core structures by means of different, complementary reactions (Figure 4, panel c). For instance, a common aryl bromide intermediate was subjected to palladium-catalyzed hydrocarbonylation to give spiro-fused class 1 spiroindolyl-indanone. Indolyl and indanone NP fragments are found in various NPs. Class 1 was further reduced to indoline class 2 which was *N*-functionalized to yield class 3. Alternatively, deprotonation and *N*-acetylation of class 1 yielded class 4, which contains an exomethylene group. A common structure found in many biologically significant alkaloids is the isoquinoline fragment, which can be attached to spiroindolyl-indanone class 1 via spiro-edge fusion, resulting in the formation of indoline-indanone-isoquinoline class 5. To obtain greater skeletal diversity, the common starting framework was rearranged to indoline-3-ones which yielded additional compound classes by means of Pd-catalyzed transformations. Thus, class 6 was obtained through intramolecular Pd-catalyzed C–N coupling in the absence of CO, whereas in the presence of CO, intramolecular aminocarbonylation resulted in PNP class 7. Lastly, tetrahydropyran fused indoles with an aryl bromide attached to the oxacycle underwent dearomative carbonylation in the presence of a Pd catalyst to form class 8. All classes are new PNP types with diverse connectivity types and NP fragment combinations which are also endowed with diverse bioactivities.^42^

## 3 Examples for PNP Design, Synthesis, and Analysis for Biological Activity

Below we describe selected examples to illustrate the design principles described above. We also show the synthesis routes to these PNPs to illustrate that PNPs are readily accessible in a few steps. For the selected examples, the bioactivity is also discussed. Further recent PNP examples from our laboratories and PNP classes reported by other laboratories since 2020 with explicit reference to the PNP principle are summarized in Table 1.

**Table 1:**
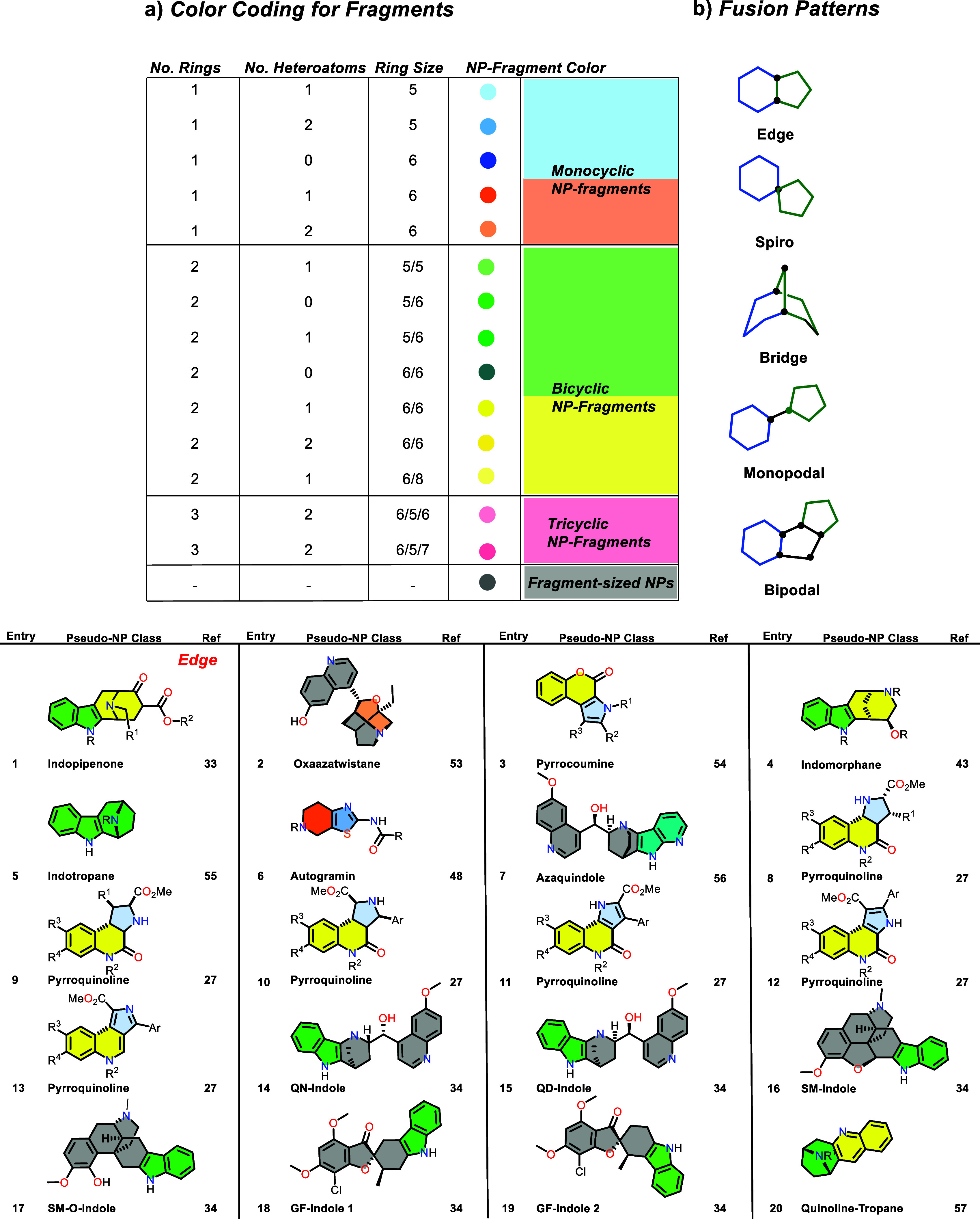

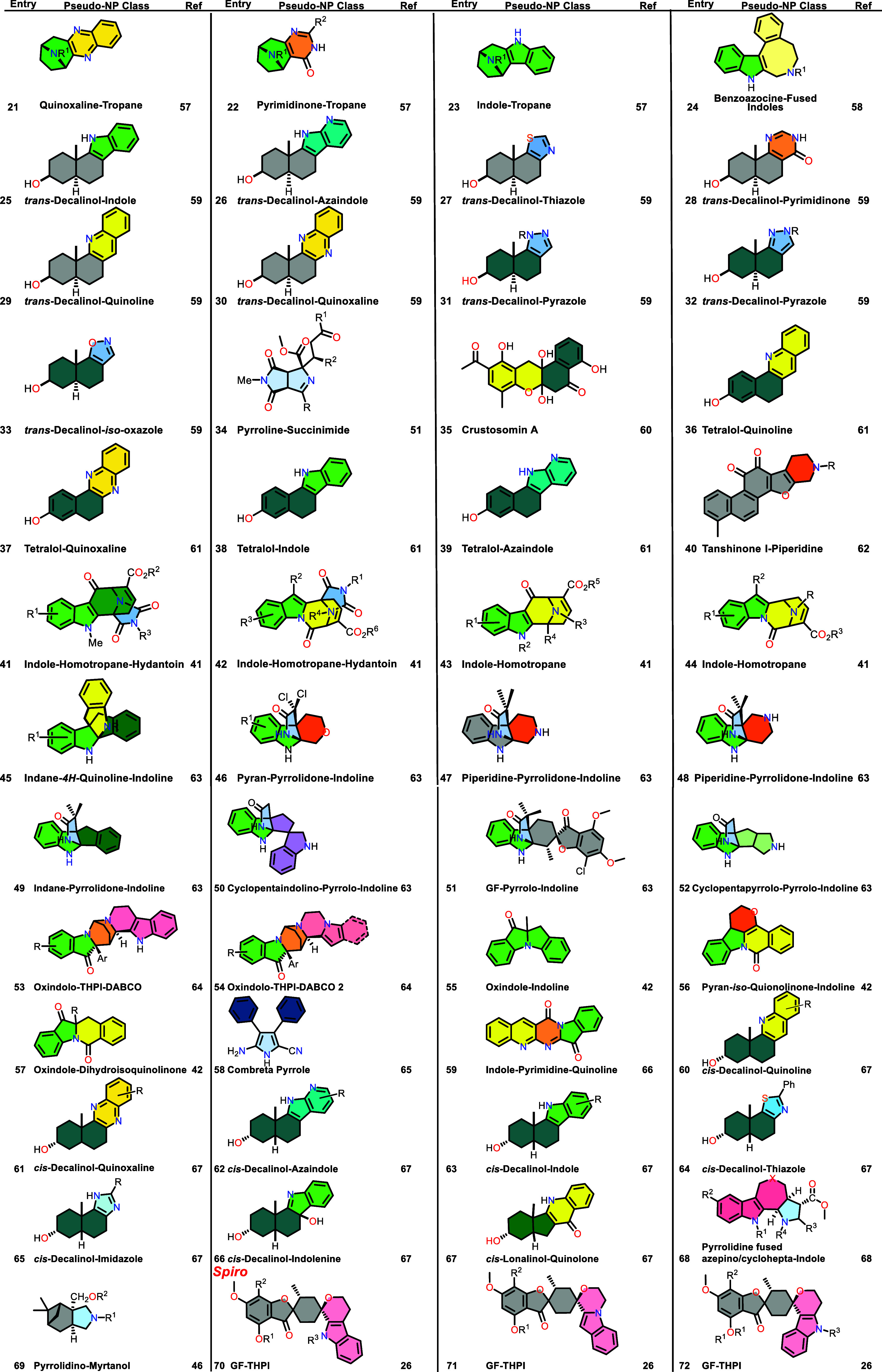

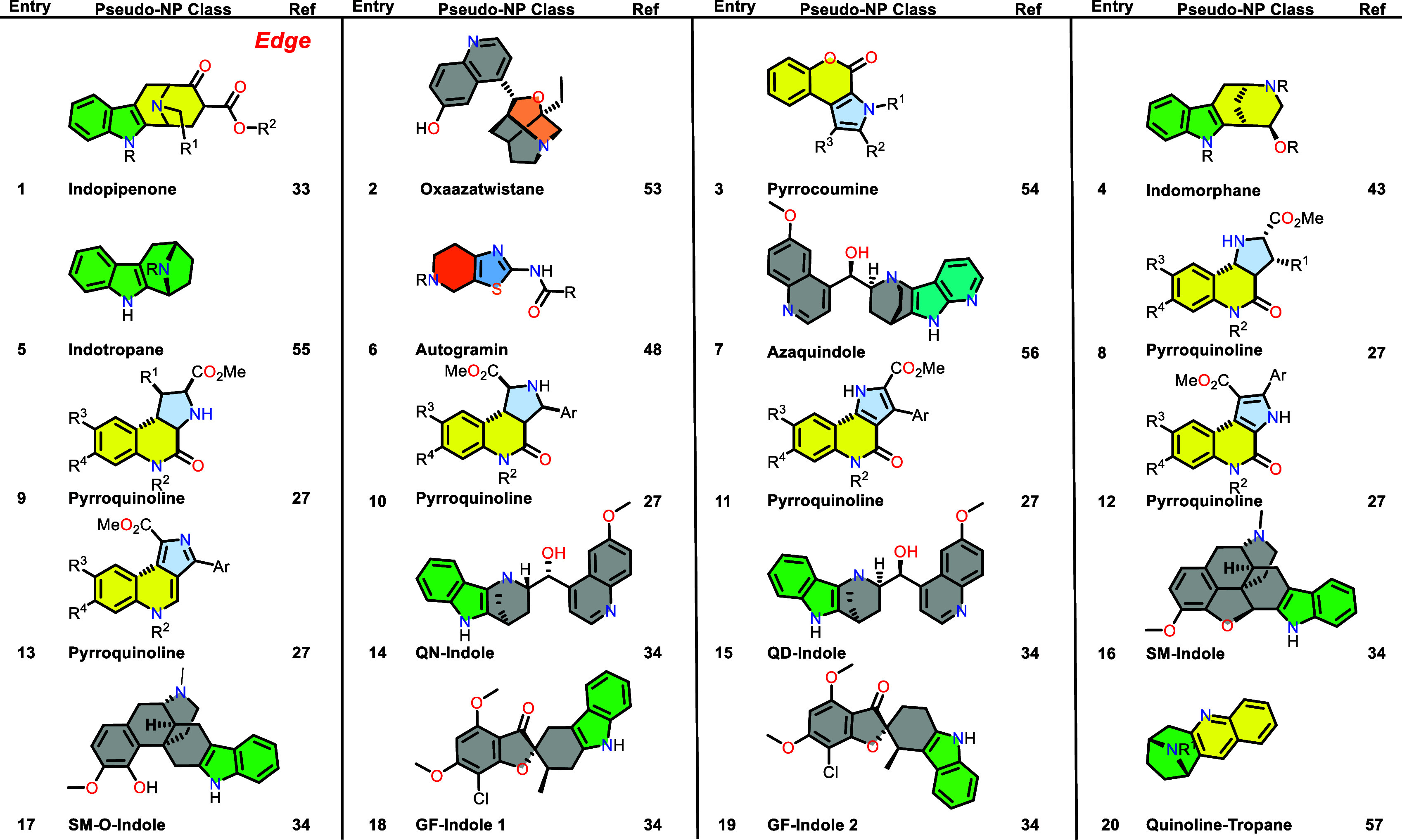
Pseudonatural Product Classes Synthesized in Our Laboratories and Reported by Other Research Groups since the Inception of the PNP Principle,^12,13–22,29^ with Explicit Reference to the PNP Approach^g^

a Entries 123 and 124 are stereoisomers.

b Four monopodal and two edge fusions.

c Spiro and bridge fusion.

d Edge and monopodal connection.

e Two edge and one bridge fusion.

f Spiro and edge fusion.

g A) Color coding for individual fragment types. Colors indicate structural features such as the number of heteroatoms in a fragment, the number of rings, and ring size. B) PNPs are categorized according to the connectivities described in Section 2, Figure 2 using fusion patterns like edge, spiro, bridge, monopodal, and bipodal. GF = griseofulvin, QN = quinine; SM = sinomenine, sm-O = sinomenine opened, DABCO = 2,6-diazabicyclo-[2.2.2]­octanes, THPI = tetrahydropyran-indole.^53–74^

### 3.1 PNPs Defining Novel Chemotypes for Targets with Existing Small Molecule Modulators

#### 3.1.1 Dual Inhibition of Glucose Transporters GLUT-1- and -3 by Indomorphans

Indole and morphan alkaloids are synthesized in nature through unrelated pathways and have different bioactivities. Merging characteristic fragments of these NP classes yielded bridged bicyclic indomorphan PNPs. Both alkaloid classes embody the piperidine fragment in different arrangements, e.g., in ergoline **40** close to the indole ring, and morphine **41**, connected to a benzene ring through a bridge in the morphan scaffold. In indomorphans **42,** the morphan fragment including the piperidine is edge fused to the indole scaffold to give the bridged bicyclic indolylethyl amine which defines the PNP class (Figure 5, panel a).^43^ For the synthesis of indomorphans, known bicyclic ketomorphan **43** was O-alkylated to yield ethers **44**, which were then subjected to Fischer indole synthesis yielding the indomorphan scaffold **45**. Subsequent indole *N*-alkylation to **46** followed by deprotection and acylation of the morphan nitrogen yielded indomorphans **47** (Figure 5, panel b).

**Figure 5:**
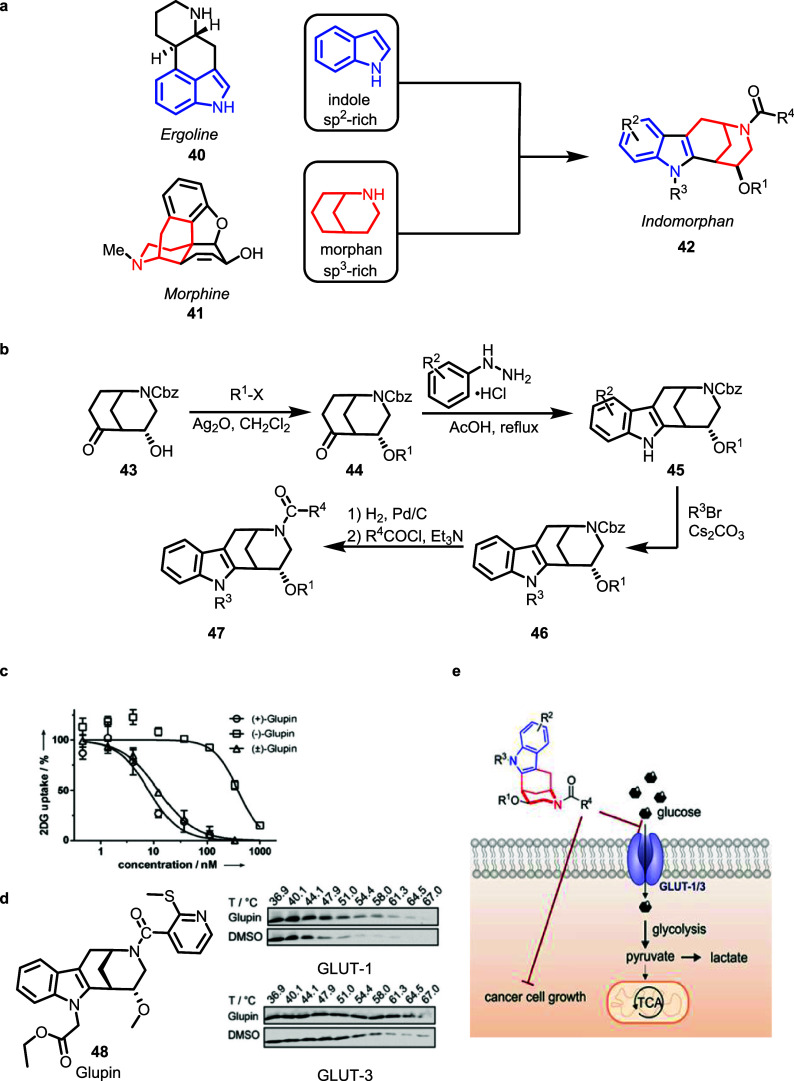
Design, synthesis, and biological evaluation of indomorphan PNPs. **a)** Design of indomorphan-PNPs combining the NP-fragments of morphan and indole. **b)** Synthesis of indomorphans. **c**) Concentration dependent 2DG uptake in the presence of different glupin stereoisomers. **d)** Cellular thermal shift assay (CETSA) to determine stabilization of glucose transporters by Glupin. **e)** Inhibition of glucose uptake and cancer cell growth through inhibition of GLUT-1/3 by Glupin. (c–e) Adapted from Ceballos et al.^43^ under CC-BY 4.0.

Investigation of the indomorphans in various cell-based assays revealed that these PNPs inhibit glucose uptake through glucose transporters GLUT-1 and -3. Increased glucose uptake is characteristic for the so-called Warburg effect, i.e., tumor cells switch their metabolism to aerobic glycolysis to fuel biopolymers synthesis, and to this end, they upregulate GLUT-1 and -3. Targeting glucose uptake has been actively pursued in anticancer drug discovery, yielding mostly polycyclic aromatic compounds as GLUT inhibitors.^44^ A cell-based assay monitoring uptake of 2-deoxyglucose revealed that Glupin-1 (**48**) potently inhibits glucose uptake in the highly glycolytic human breast cancer cell line MDA-MB-231 (IC_50_ = 4 ± 2 nM) (Figure 5, panel c). Investigation of the compound in a cellular thermal shift assay (CETSA) demonstrated stabilization of GLUT-1 and -3 by the PNP, which proved target engagement (Figure 5, panel d). Inhibition of the activity of both transporters is crucial to impair growth of cancer cells (Figure 5, panel e), and Glupin-1 displayed highly selective and potent activity against cancer cells that depend on glucose uptake for growth.

#### 3.1.2 Tafbromin Targets Bromodomain 2 of Transcription Factor TAF1

Biosynthetically unrelated, pyrrolidine- and tetrahydroquinoline alkaloid fragments present, for instance, in kainic acid **30** and virantmicin **49** were successfully fused in different arrangements to pyrroquinoline (PQ) PNPs. Notably, PQ bioactivity profiles varied depending on the connectivity of the combined fragments.^27^ The design involved the fusion of the pyrrolidine to the g-side of the tetrahydroquinoline resulting in the formation of the PQ scaffold **53** (Figure 6, panel a).^45^

**Figure 6:**
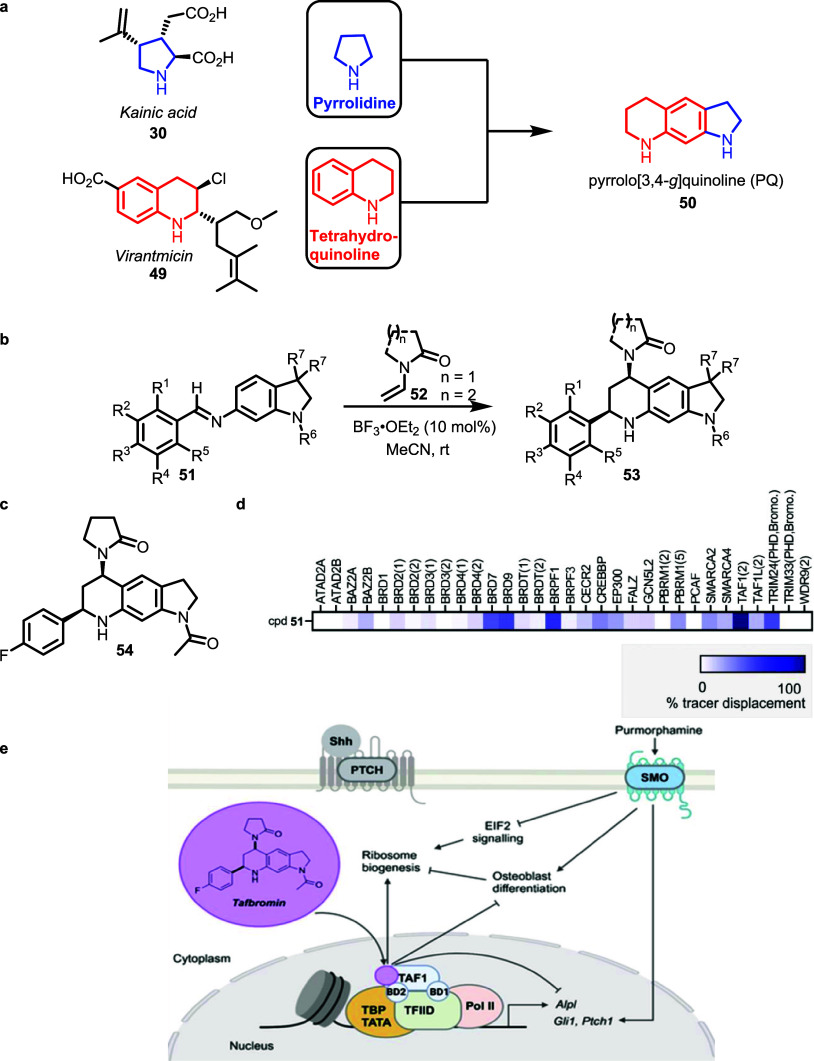
Design, synthesis, and biological evaluation of pyrroquinoline PNPs. **a)** Design of PQ PNPs **53** by combining NP fragments, pyrrolidine of kainic acid, and tetrahydroquinoline of virantmicin. PNPs with structure **50** contain an edge fusion of an aromatic ring and an aliphatic ring. **b)** Synthesis of PQ utilizing Lewis acid catalyzed Povarov reaction. **c)** Alpl gene expression declines in the presence of 1.5 μM purmorphamine (antagonist of Hh signaling pathway) and increasing Tafbromin (cis-diastereomer of **54**) concentrations. **d)** BROMOscan panel profiling for isomeric mixture **54** at 10 μM. Data are percent inhibition of tracer binding to the respective bromodomain. **e)** Tafbromin exerts its inhibitory effect on osteoblast differentiation by selectively binding to BD2 of TAF1. This interaction reduces the expression of Hedgehog target genes Gli1 and Ptch1 and the osteogenic marker Alpl. Consequently, osteogenesis is suppressed, overcoming the inhibition of ribosome biogenesis. Figure 6e and caption 6d adapted from Patil et al.^45^ CC-BY-NC-ND 4.0.

The pseudonatural product class **53** was synthesized by means of a Povarov reaction of indoline-derived Schiff bases **51** with electron-rich vinylpyrrolidone dienophiles **52** (Figure 6, panel b). Investigation of these PNPs in a variety of phenotypic assays which monitor various biological processes e.g., signaling cascades, autophagy, and glucose uptake, revealed that PQs affect the Hedgehog signaling pathway, which is essential for the development of vertebrate embryos and for maintaining adult cell balance. Binding of a Hedgehog ligand, e.g., sonic hedgehog (Shh), to the transmembrane receptor patched-1 (PTCH1) releases inhibition of the protein smoothened (SMO), and subsequent translocation of glioma-associated homologue (GLI) transcription factors into the nucleus activates expression of Hedgehog target genes, like *Gl1l* and *Ptch1*. In the cell-based assay, Hedgehog signaling was activated by the SMO agonist Purmorphamine and monitored as the expression of the osteoblast marker alkaline phosphatase in the pluripotent murine mesenchymal stem cell line C3H10T1/2. *para*-F PQ **54** displayed an IC_50_ of 0.9 ± 0.2 μM and dose-dependent reduction of the alkaline phosphatase *Alpl* gene expression. Bioinformatics analysis utilizing a polypharmacology browser suggested that the compound, coined Tafbromin (*cis* diastereomer of **54**), reduces Hh target gene expression during osteoblast differentiation by selectively targeting the second bromodomain of the transcription activator TAF1 (Figure 6, panel d). TAF1 is the largest constituent of the basal transcription factor IID (TFIID). It controls cell cycle genes and stem cell reprogramming, and its mutation is implicated in various cellular processes such as cell growth, differentiation, and disease. This multidomain protein also embodies two bromodomains (BD1 and BD2), and in-depth analyses revealed that Tafbromin is a nontoxic, selective TAF1 (2) ligand that promises to be a valuable tool for exploring TAF1-related biology (Figure 6, panel e).

#### 3.1.3 iDegs Induce Degradation of IDO1 Mediated by KLHDC3

Edge fusion of the fragment-sized bicyclic monoterpenoid myrtenol **55** with the pyrrolidine fragment, frequently found in alkaloids such as nicotine **29** yielded pyrrolidino-myrtanol PNPs **56** (Figure 7, panel a).^46^ Edge-fusion to **59** was achieved by means of a regio- and stereoselective [3 + 2] cycloaddition involving an azomethine ylide derived from *N*-(methoxymethyl)-*N*-(trimethylsilylmethyl)­benzylamine **57**, and the terpenoid alkene **58** as the dipolarophile (Figure 7, panel b). Subsequent reduction yielded primary alcohol **60** which was carbamoylated to **61**. Finally, one-pot *N*-benzylation and sulfonamide formation led to iDegs **62**. These PNPs inhibited the formation of the immunosuppressive metabolite kynurenine produced from tryptophan by indoleamine-2,3-dioxygenase 1 (IDO1) upon stimulation with cytokines like IFN-γ secreted, for instance, from tumor cells. This immunomodulatory process hides the tumor from the immune system, and inhibition of IDO1 is pursued as a novel approach to anticancer drug discovery.^47^ iDeg-1 **63** reduced Kyn production in BxPC3-cells with an IC_50_ value of 0.83 ± 0.31 μM without affecting enzyme activity, transcription, or translation (Figure 7, panel c). Structure optimization led to the identification of the more potent iDeg-6 **64** which has an IC_50_ of 16 ± 5 nM in the Kyn assay (Figure 7, panel d). iDeg-6 both inhibited IDO1 and induced degradation of the protein in cells with a *D*_max_ of 70% at 100 nM and DC_50_ of 6.5 ± 3 nM (Figure 7, panel e). Surprisingly, degradation of IDO1 occurs through the ubiquitin-proteasome system by recruiting the KLHDC3 E3 ligase, which had not previously been associated with small molecule-induced protein degradation. In fact, KLHDC3 is one of the natural ligases targeting IDO1, such that iDeg-6 binding accelerates the natural degradation pathway. Mechanistically, iDegs bind to the heme binding site in the apo-form of the enzyme. As opposed to other apo-form binders, they appear to establish a complex structure that is more prone to degradation than the heme-bound holo form (Figure 7, panel f). Thus, iDegs define a novel monovalent degrader chemotype, and their mode of action includes supercharging of the native IDO1 degradation pathway. iDeg-1 binding to IDO1 was confirmed by means of a thermal shift assay (CETSA) which demonstrated protein stabilization of ΔTm = 3.5 ± 0.4 °C. iDegs on the one hand can be considered as a novel inhibitor chemotype for a target with existing inhibitor classes. However, on the other hand, they can also be viewed as the first small molecules that induce degradation of IDO1, i.e., for a target with no other degraders identified before.

**Figure 7:**
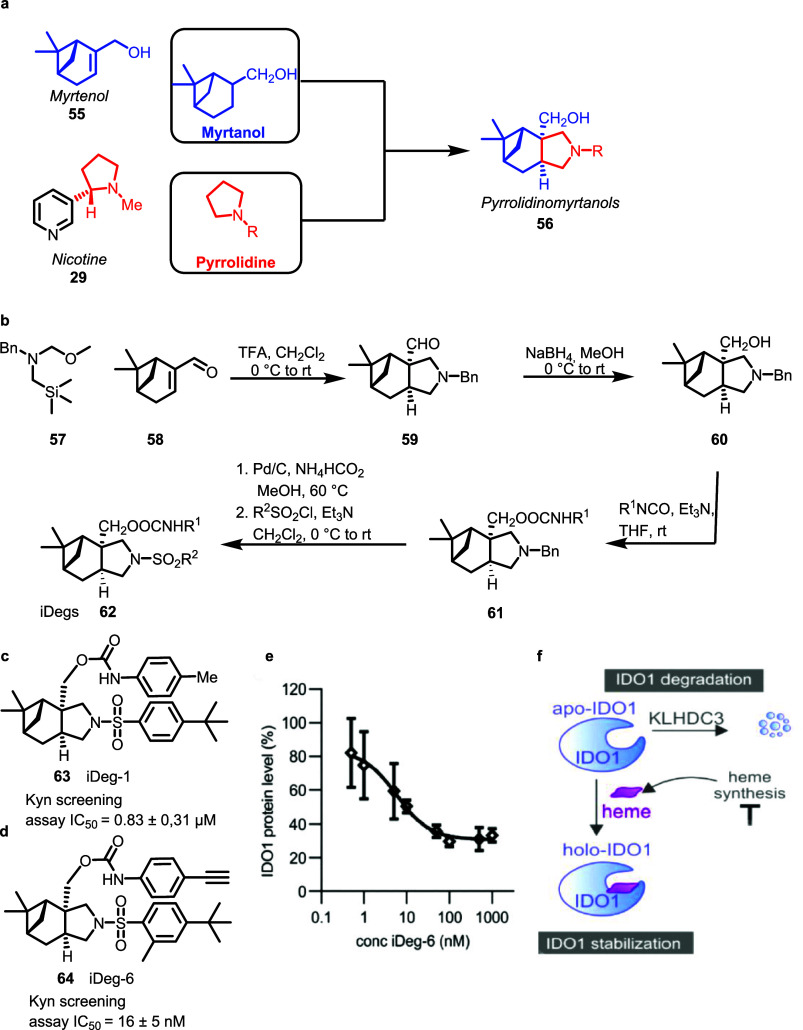
Design, synthesis, and biological evaluation of pyrrolidino-myrtanol PNPs. **a)** Design of pyrrolidino-myrtanol PNPs. **b)** Synthetic route to iDegs **62** utilizing the [3 + 2] cycloaddition. **c)** Structure of screening hit iDeg-1 and IC_50_ value in the Kyn assay in BxPC3 cells. **d)** iDeg-6 Kyn screening with corresponding result from the Kyn assay. **e)** Reduction of IDO1 protein levels through iDeg-6. **f)** Regulation of IDO1 by heme. Figure 7e–f adapted from Hennes et al.^46^ under CC-BY-ND 4.0.

### 3.2 PNPs Defining Chemotypes for Targets without Existing Small Molecule Modulators

#### 3.2.1 Autogramin PNPs Target and Reveal the Role of Cholesterol Transport Protein GRAMD1A in Autophagosome Biogenesis

Thiazole and piperidine fragments occur in alkaloids, e.g., bacillamide **65** and anabasine **66,** and can be combined to piperidinothiazole PNPs **67** (Figure 8, panel a). The synthesis involves α-bromination of piperidin-4-one **68** with dibromo barbiturate **69** to yield **70**. Subsequent cyclization leads to piperidinothiazole core structure **71**, which can be coupled in monopodal manner with diverse aromatic acids **72** to **73** (Figure 8, panel b) which themselves can be NP fragments or close analogs thereof (e.g., the piperazinedione embedded in Autogramin-1).^48^ An unbiased phenotypic screen monitoring autophagy identified the aminothiazoles as new autophagy inhibitors (Figure 8, panel c).^49^ Autophagy is central to the maintenance of cellular homeostasis. Autophagy involves formation of autophagosomes that engulf damaged macromolecules, protein aggregates, and organelles, and after fusion with the lysosome, this cargo is digested and recycled. Misregulation of autophagy is a hallmark of different diseases.

**Figure 8:**
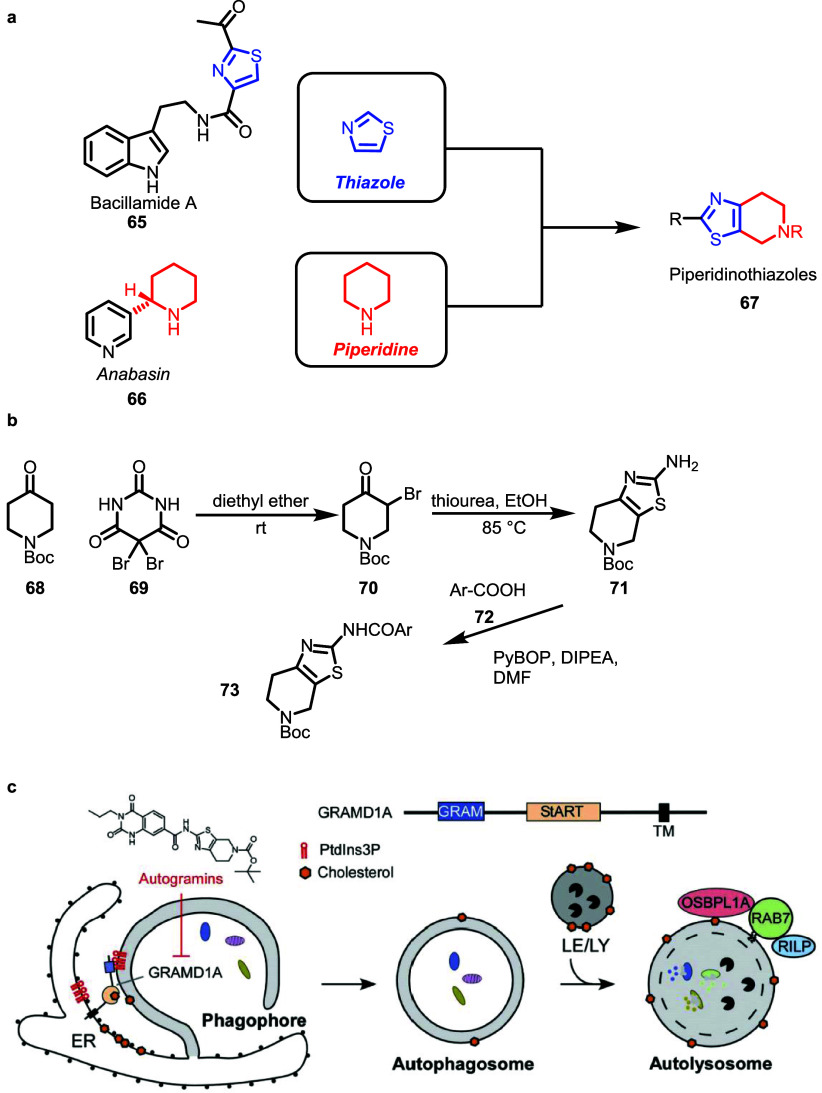
Design, synthesis, and biological evaluation of piperidinathiazole PNPs. **a)** Design of piperidinothiazoles. PNPs with structure **67** contain an edge fusion of an aromatic and an aliphatic ring. **b)** Synthetic route toward amino piperidinothiazoles. **c)** Role of GRAMD1A and cholesterol in autophagosome formation: enrichment of PtdIns3P at the autophagosome initiation sites also increases levels of GRAMD1A near the endoplasmic reticulum (ER). GRAMD1A mediates cholesterol transport between the organelles and, therefore, promotes autophagosome biogenesis. Figure 8c adapted from Wu and Waldmann.^49^

Autophagy is activated in response to cellular stress, such as nutrient deprivation, which triggers the formation of a phagophore and eventually the autophagosome which fuses with a lysosome to the autolysosome, where the cellular components are degraded.^50^ Target identification by means of affinity enrichment and proteomics and target validation by means of different methods, including a cellular thermal shift assay, revealed the protein GRAMD1A as target of the PNPs which, hence, were termed autogramins.

Subsequent in-depth characterization of the mode of action revealed that autogramins interfere with the transfer of cholesterol between membranes during early stages of autophagosome formation, mediated by the cholesterol transport protein GRAMD1A. This insight into autophagy modulation through inhibition of cholesterol transfer and binding, established GRAMD1A as a new protein involved in autophagy regulation.^48^

#### 3.2.2 The PNP Rhonin Targets RHOGDI

NP-derived five-membered pyrrolidine-, succinimide-, and pyrroline-fragments originating from **74**–**76** and **29** (Figure 9, panel a) were combined by means of edge fusion as in **77** and **78**, a combination of three fragments including an edge fusion and a monopodal connection, as in **79**, and a combination of edge fusion and bicyclic connection to bridge fusion **80** to yield a PNP collection. The synthesis involved asymmetric 1,3-dipolar cycloaddition between maleimides **81** and azomethine ylides **82** to **83** (Figure 9, panel b), followed by oxidation to imines **84** which were subjected to different transformations resulting in the formation of compound classes **85**-**87**.^51^ Target-agnostic phenotypic assays identified library member **88a** that inhibits osteoblast differentiation in pluripotent mesenchymal C3*H*/10T1/2 mouse cells through interference with the Hh pathway (see also above). However, the compound did not inhibit the orthogonal GLI-dependent reporter gene assay in Sonic hedgehog (Shh)-LIGHT2 cells but partially suppressed the expression of the Hh target genes *Ptch1* and *Gli1* (Figure 9, panel e). In contrast to the majority of established Hh pathway inhibitors, compound **88a** did not target the transmembrane protein smoothened (SMO). Affinity isolation employing probe **89a** identified RHO-GDP-dissociation inhibitor 1 (RHOGDI1) as the cellular target of the PNP (Figure 9, panel f), such that the compound was termed Rhonin. RHOGDI1 is a cellular chaperone for geranylgeranylated Rho GTPases and stabilizes them in solution (Figure 9, panel d), and **88a** directly binds to the geranylgeranyl binding site of RHOGDI with low micromolar affinity. The GTP-binding RAC protein, which belongs to the Rho GTPase family, binds to RHOGDI *in vitro* with a *K*_d_ of 5.7 μM. RHOGDI acts as a negative modulator of RHO GTPases, and the inhibitory effect of Rhonin increases the activity of GTP-bound RHO GTPases. Additionally, treatment with Rhonin causes a redistribution of membrane-bound RHO GTPases, such as RHOA and RAC1, toward the endoplasmic reticulum membrane, which subsequently interferes with Hh signaling in a noncanonical manner.

**Figure 9:**
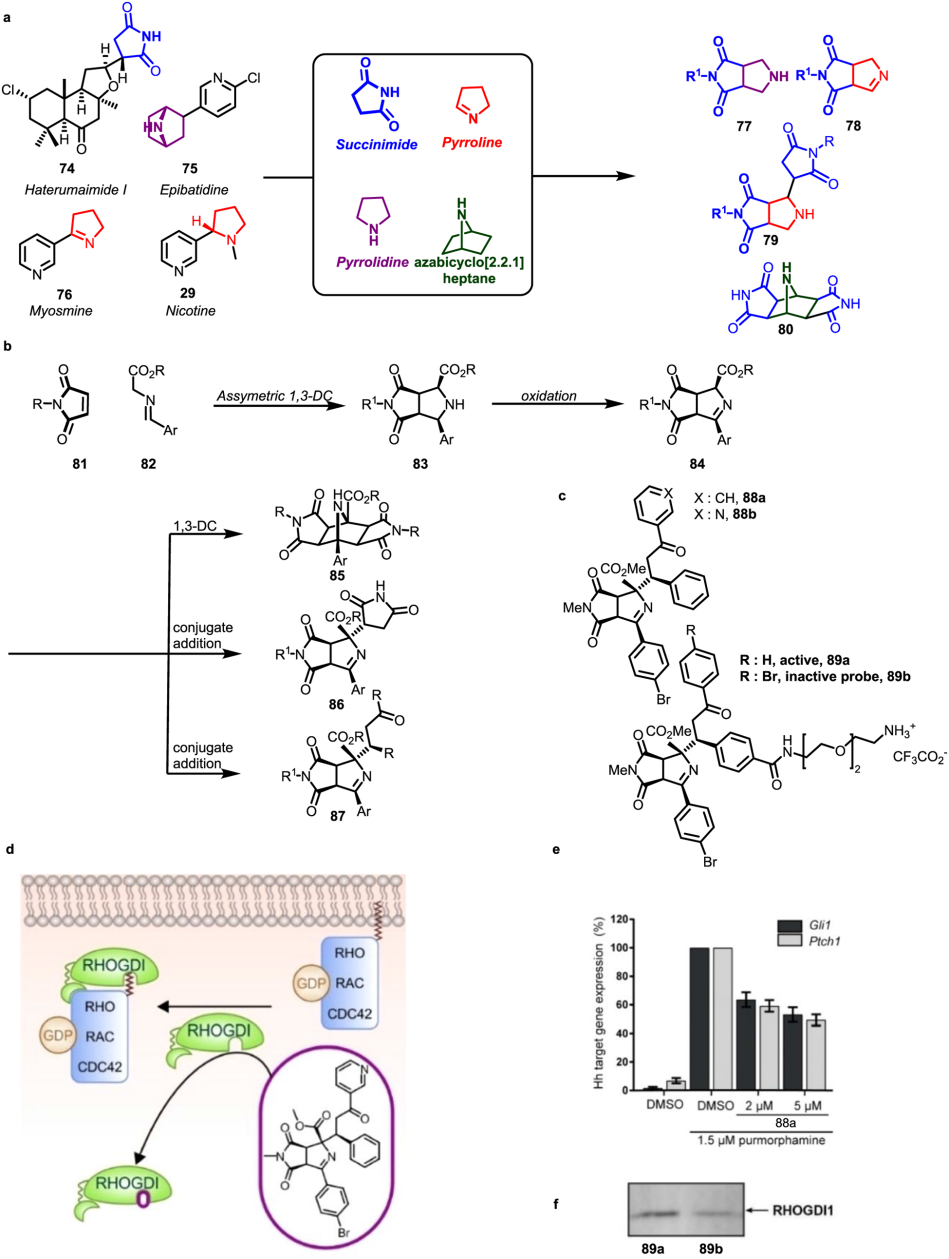
Design, synthesis, and biological evaluation of pyrrolidino- and pyrroline succinimide PNPs. **a)** Design of diverse pyrrolidino-succinimide PNPs. **b)** Synthetic route toward various polypyrrolidines. **c)** Target identification of Rhonin with samples **88a** and **88b** and affinity probes for the pull-down experiment **89a** and **89b**. **d)** Proposed mode of action for Rhonin **88b. e)** Downregulation of *Gli1* and *Ptch1*. **f)** Affinity-based enrichment of RHOGDI1 by affinity probe **89a** is active compared to inactive **89b**. Figure 9d–g modified and adapted from Akbarzadeh et al.^51^ under CC-BY 4.0.

## 4 Different Combinations and Arrangements of NP Fragments Yield Chemically and Biologically Diverse Compound Collections

Different combinations and arrangements of a particular set of NP fragments to give PNPs are expected to yield biologically and chemically diverse PNP classes which display novel bioactivities that differ from the activity of the originating NPs. This notion was verified by the design, synthesis, and biological analysis of a PNP collection in which the highly NP-prevalent indole or chromanone ring systems were fused with fragments derived from fragment-sized Cinchona alkaloids quinine (**QN**) and quinidine (**QD**), griseofulvin (**GF**), and sinomenine (**SM**) (Figure 10).^34^ To this end, ketone fragments were synthesized from NPs and then subjected to different annulation reactions. Edge fusion to form an indole was achieved through the Fischer indole synthesis and palladium-catalyzed annulation and yielded PNP classes **QN-I**, **QD-I**, and **GF-I**, as well as **SM-** PNPs with a closed heptacyclic structure (**SM-I-closed**) or with one ring opened (**SM-I-open)**. Oxa-Pictet–Spengler reactions and Kabbe condensations were employed to synthesize spiro-fused PNPs. Regioisomers formed in the indolisations and diastereomers at the spirocyclic point of fragment condensation formed during the Kabbe condensation could be separated in several cases. Overall, a library of 244 PNPs was prepared which can be categorized into 8 classes and 13 subclasses which represent different connectivity patterns, regio- and stereoisomers.

**Figure 10:**
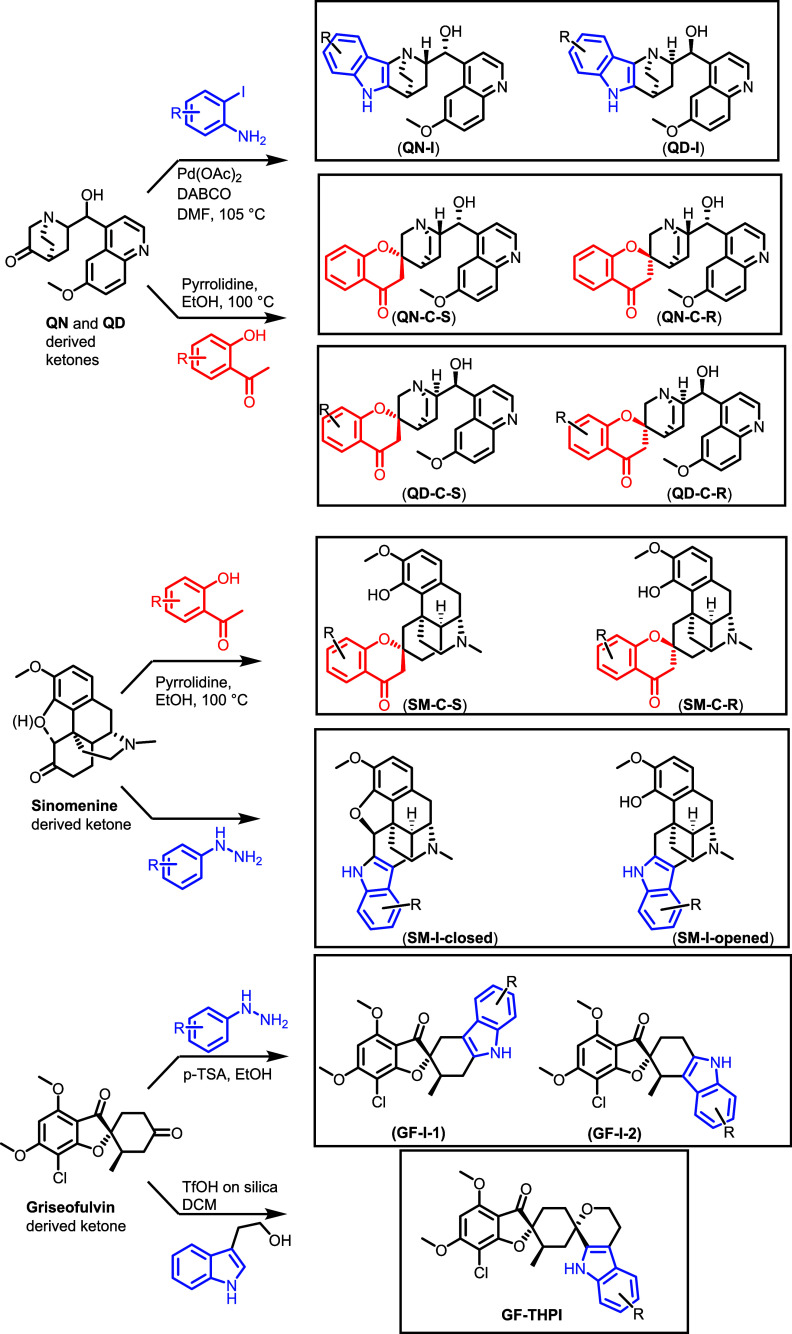
Classes of pseudonatural products derived from ketone derivatives of fragment-sized NPs. PNPs **QN-I**, **QD-I,** and **GF-I** contain an edge fusion of an aromatic and an aliphatic ring. Figure 10 modified from Grigalunas et al.^34^ under CC-BY 4.0.

Cheminformatics analysis of the compounds revealed that different combinations of a small set of NP fragments yielded a chemically diverse library with homogeneous subclasses. Median Tanimoto similarity of the Morgan fingerprints was 0.75 within the 13 subclasses (Figure 11, panel a), but intersubclasses median similarity was only 0.26 in cross-subclass comparisons (Figure 11, panel b).

**Figure 11:**
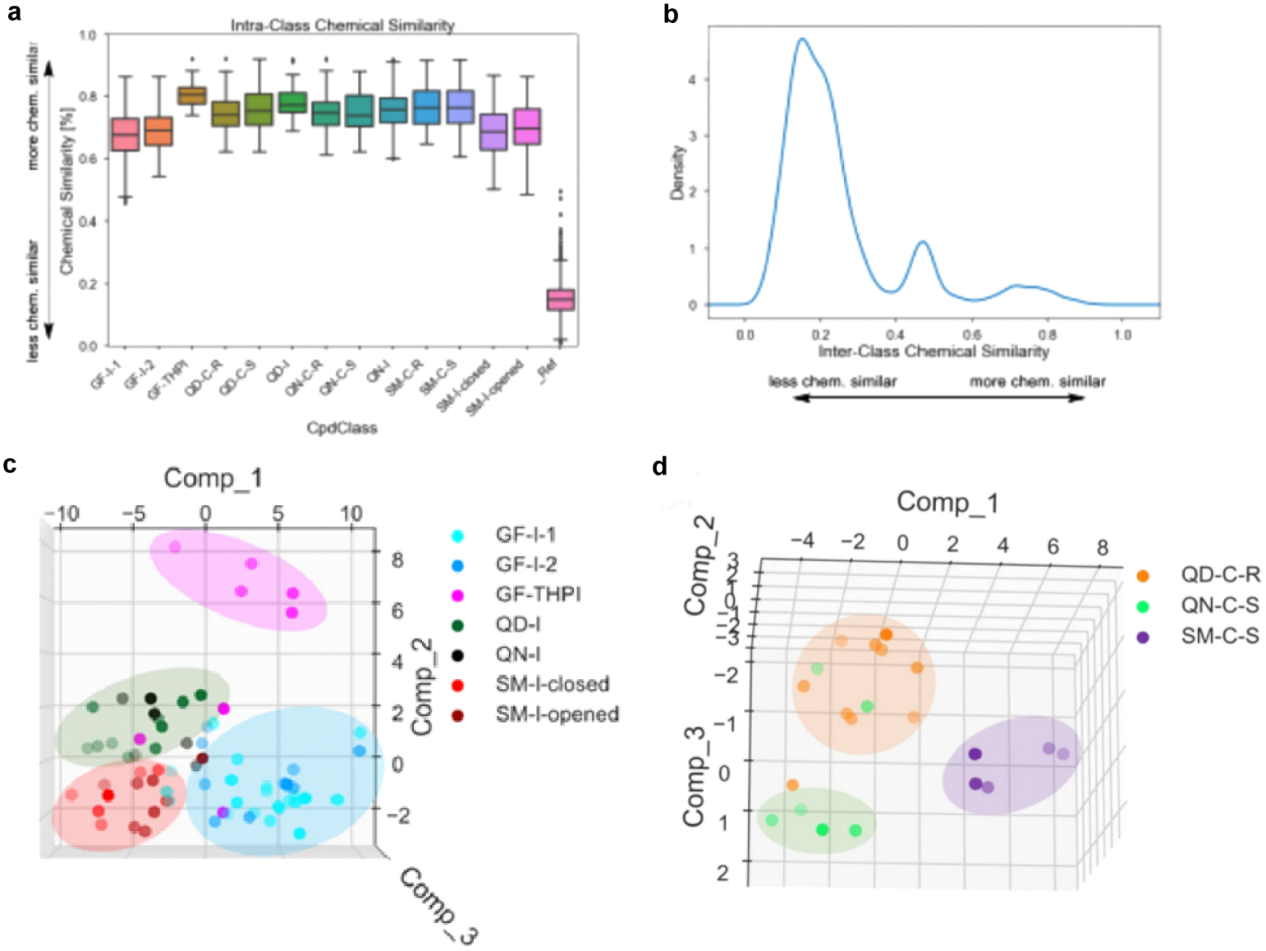
Tanimoto similarities of Morgan fingerprints of the PNP library. **a**) Intrasubclass comparisons. **b**) Intersubclass comparisons. **c**) PCA of indole containing PNPs. **d**) PCA of chromanone containing PNPs. Figure 11a–d adapted from Grigalunas et al.^34^ under CC-BY 4.0.

The bioactivity and biological diversity of the synthesized PNP library were assessed through morphological profiling using the cell painting (CP) assay,^52^ which monitors bioactivity in a broad sense. In this multiparametric assay, six fluorescent dyes are employed to capture morphological details with multichannel fluorescent microscopy, examining 579 reproducible features to create characteristic profiles.

Phenotypic activity was quantified by calculating the number of significantly altered features resulting in an induction score (in %). Similarity among phenotypic profiles was assessed through correlation distances, represented as biosimilarity percentage, where profiles with 75% or higher are classified as biosimilar. Cross-similarity analysis and calculation of a median biosimilarity percentage (MBP) facilitated the comparison of the compound classes. Principal component analysis (PCA) was utilized to visually represent the differences and similarities among classes in three-dimensional plots. The formation of clusters in PCA indicates significant phenotypic differences, while the absence of clusters suggests phenotypic similarity. The analysis revealed the high bioactivity of the PNP library. In total, 84% of the synthesized PNPs induced significant morphological changes (induction >5%), the median induction at 10 μM was 17%, and induction values ranged from 5 to 70%. Cross similarity analysis of the phenotypic characteristics of the sublibraries revealed a high similarity (MBP of 77%) within the subclasses, while the interclass similarity was relatively low (MBP of 61%). This difference is in close analogy to the findings of the cheminformatics analysis of the PNP library.

In depth analysis showed that different fragment combinations, stereochemistry, and connectivity patterns have a profound phenotypic effect resulting in significant differences in phenotypic profiles among the respective subclasses. In order to determine whether different combination of unrelated fragments led to different bioactivities, sublibraries containing either an indole- or chromanone fragment were analyzed and the common fragments within the PNPs were classified as nondominating (PNPs containing the same fragment cluster in PCA and/or have a low MBP < 75%) or dominating (compounds that do not cluster in PCA and have a high MBP are dominating). Fragments which do not dominate bioactivity of the new combination can be considered favorable choices for the design and synthesis of further PNP classes with novel fragment combinations.

Classification and combination of nondominating and dominating fragments could guide synthesis design to yield biologically diverse collections. In PNPs representing combinations of nondominating fragments, bioactivity profiles likely are neither a representation of either individual fragment nor the addition of both individual fragments’ profiles, but rather the new profile may reflect the combination of fragments. Other combinations of nondominating fragments may thus lead to different biological profiles. However, if a dominating and a nondominating fragment are combined, the new profile may already be represented by the dominating fragment in other combinations. Identification and exclusion of dominating fragments will avoid compounds with redundant profiles.

Cluster analyses revealed that all indole clusters were well-separated, and the MBP between indole-derived subclasses is low (45%) which suggests that the indole fragment is nondominating (Figure 11, panel c). Comparison of the chromanone clusters **QD-C-R**, **QN-C-R**, and **SM-C-R** suggested that also this fragment may be nondominating, as indicated by PCA separation, despite an ambiguous MPB of 74% (Figure 11, panel d). By analogy, griseofulvin and quinidine were classified as nondominating, while sinomenine emerged as a dominant fragment in PCA classification.

The notion that linking nondominating fragments should yield novel PNP classes with unique bioactivity profiles was explored by combining the nondominating indole- and chromanone fragments to yield a novel PNP class for which MBP, when compared to the indole and chromanone sublibraries, was only low (44 and 42% respectively) and which also clustered separately in the PCA.

## 5 Occurrence in Bioactive Compounds and Commercial Availability of PNPs

The examples described above provide proofs-of-principle for the PNP concept. In addition, cheminformatics analysis has indicated that large screening libraries often are biased toward biogenic molecules, i.e., NPs and related compounds.^75^ These observations and analogous findings for our in-house compound collection (we had, in fact, already synthesized various NP classes in the context of previous BIOS programs) suggested that PNPs might have been synthesized and subjected to biological analysis before, without explicit inspiration by the PNP principle, but rather by chemically intuitive use of NP-derived structures in compound collection design.

Indeed, analysis of the ChEMBL database version 32 (v32), a large collection of mainly synthetic compounds and their activities, by means of a newly developed cheminformatic NP fragment combination (NPFC) analysis tool revealed that PNPs already constitute a significant fraction of currently known bioactive compounds.^76^

The NPFC tool employs 1673 NP fragments, derived from the analysis by Over et al.,^20^ and identifies naturally occurring fragment combinations by comparison with a reference NP data set, listed for instance in the dictionary of natural products (DNP). The fragment combinations identified in the data set of interest (ChEMBL database)^77^ are then compared with the fragment combinations found in the NP reference set of the DNP,^78^ and by parsing the output of the tool compounds, they are consequently assigned to one of four categories:

***NP***, if a structure is identical to an NP,

***NonPNP***, if the structure does not contain any valid fragment combination or if it was removed by a preprocessing filter,

***NPL*** (NP-like), if a structure only contains fragment combinations that are already known from NPs, i.e., naturally occurring, and finally,

***PNP***, if a structure contains NP fragment combinations that are not occurring in the reference NP data set.

For the PNPs, the tool also extracts the types of fragment combinations, enabling a statistical analysis of the most frequent fragments and their combination types in the investigated data set.

NPFC analysis for ChEMBL v32 (2.3 million structures, released 2023) identified 690,000 PNPs out of 2.1 M compounds in total (32%; deduplicated by the InChIKeys of the racemized structures).^36^ This finding demonstrated the applicability of the PNP approach in drug discovery, given the identification of widespread reports of bioactivity within these compounds.

Among the ChEMBL PNPs, the linear connection (connection monopodal (cm)) is the most prevalent (74%), followed by an edge fusion (fusion edge (fe)), a bridge fusion (fb), spiro fusion (fs), and the bipodal edge connection (cbe), with relative distributions of cm: fe: fb: fs: cbe ≈ 100:22:5:3:2.5, amounting to 97.7% of all identified connection types (Figure 12, panel b shows graphical representations of the different connection types).

**Figure 12:**
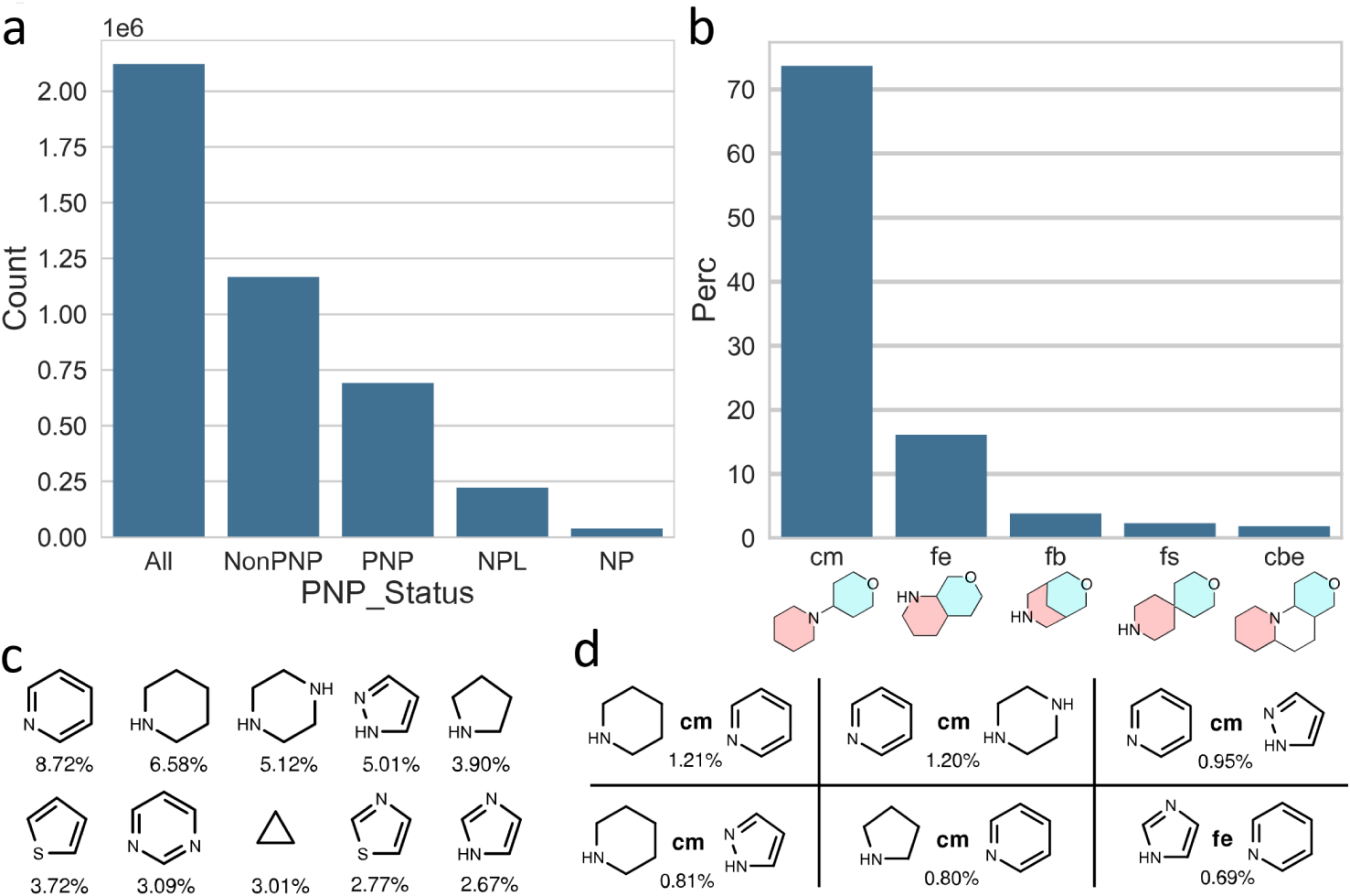
Most common NP fragments and fragment combinations identified in the ChEMBL v32 data set. **a)** Distribution of PNP status for the ChEMBL v32 data set, deduplicated by InChIKeys (*y*-axis scale in millions of compounds, 1e6). **b-d)** Data for PNP compounds, only. **b)** Distribution of most common NP fragment connection types in percent; cm: connection monopodal, fe: fusion edge, fb: fusion bridge, fs: fusion spiro, cbe: connection bipodal edge. **c)** Most common NP fragments, with percent occurrence in all PNP NP fragment combinations. **d)** Most common NP fragment combinations, with connection type and percent occurrence (see **b** for an explanation of connection types). Figure 12 and caption adapted from Pahl et al.^36^ under CC-BY 4.0.

Pyridine, piperidine, and piperazine are the most common NP fragments identified in PNP fragment combinations (8.72, 6.58, and 5.12%), whereas the monopodal connections between pyridine and piperidine or piperazine were the most common combinations overall (1.21 and 1.20%, respectively). Notably, the analysis of the ChEMBL PNPs revealed that they consist of a large number of smaller structurally different compound class collections rather than a few large compound libraries. Figure 12 summarizes the results.^79^

These findings demonstrated that PNPs, unwittingly, have been synthesized for at least 45 years and that they frequently occur in diverse bioactive compounds. They also validated the PNP principle as a historically proven general concept for the discovery of new bioactive chemical matter.

Subsequent application of the NPFC tool for analogous analysis for PNP content of the 3.5 million synthetic small molecules in the screening library from Enamine Ltd.,^80^ which is widely sourced by the scientific community,^36^ as a proxy for potential future bioactive compounds, revealed that large numbers of structurally different PNPs are readily available.

As shown in Figure 13, the analysis revealed remarkable similarity to the conclusions drawn for the ChEMBL database. Thus, 1.1 million PNP structures were identified among the collection of 3.5 million (32%), again confirming the ubiquitous and intuitive application of the PNP principle, also among early phase research compounds. The linear monopodal connection (cm) was again the most frequent and indeed even more pronounced (83%) than among ChEMBL compounds, followed by fusion edge (fe), fusion spiro (fs), fusion bridge (fb), and connection bipodal edge (cbe), amounting to 99.7% of the total identified connection types. The relative distribution of the different connection and fusion types is cm: fe: fb: fs: cbe ≈ 100:15:3.0:2.2:0.36. The most common fragments were pyrazole, pyridine, and piperidine (7.56, 7.32, and 6.80%, respectively) and the most common fragment combinations were the linear connections between pyrrole and piperidine and between pyridine and piperazine (1.70 and 1.61%, respectively).

**Figure 13:**
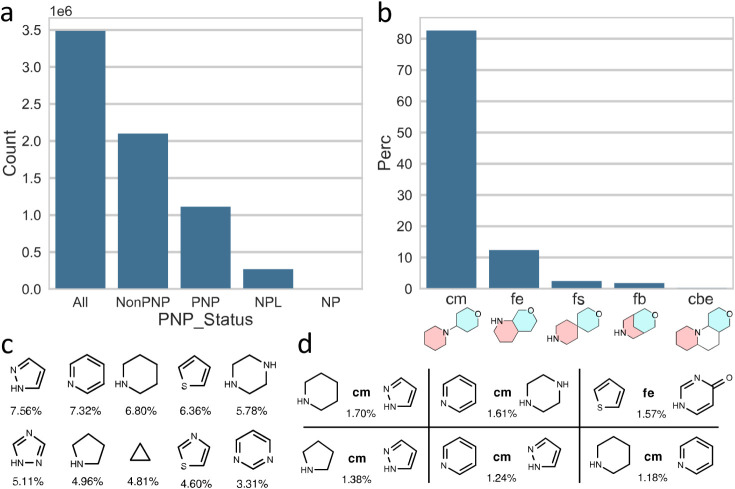
Most common NP fragments and fragment combinations were identified in the Enamine screening library. **a)** Distribution of PNP status for the full Enamine data set (*y*-axis scale in millions of compounds, 1e6). **b–d)** Data for PNP compounds, only. **b)** Distribution of most common NP fragment connection types in percent; cm: connection monopodal, fe: fusion edge, fs: fusion spiro, fb: fusion bridge, cbe: connection bipodal edge. **c)** Most common NP fragments, with percent occurrence in all PNP NP fragment combinations. **d)** Most common NP fragment combinations, with connection type and percent occurrence (see **b** for explanation of connection types). Figure 13 and caption adapted from Pahl et al.^36^ under CC-BY 4.0.

Since commercial screening compounds often serve as starting points for discovery programs, the bioactivity range for a representative selection of 875 PNPs available from Enamine, encompassing 250 diverse scaffolds, was investigated by means of the morphological cell painting assay (CPA). As mentioned above, this morphological assay broadly monitors bioactivity by selective staining of intracellular compartments and determination of hundreds of cellular parameters that are condensed into a bioactivity profile. It determines an induction value, i.e., the percentage of significantly changed features in a profile, as a measure of bioactivity, and a compound is considered to be active with an induction ≥5%.^36^ Investigation of the 875 PNP compounds from Enamine revealed that 16% of the PNPs showed significant activity at 30 μM, and even at 10 μM, 44 compounds (5%) were active. In-depth analysis for diverse bioactivity showed that among the analyzed PNPs, phenotypes characteristic of tubulin modulation, lysosomotropism/cholesterol homeostasis, DNA synthesis inhibition, mitochondrial stress, and HDAC inhibition could be identified. A significant number of the active compounds, i.e. 106 out of 143 (74%) at 30 μM and 28 out of 44 (64%) at 10 μM could not be assigned to any previously identified phenotype, which indicates novelty and diversity in bioactivity.

The hit rate observed for the compounds selected from the Enamine collection is lower than the rate typically observed for in-house PNPs and reference compounds (hit rate: 31%). The Enamine screening collection was designed with a focus on generating high-quality hits, and the molecule size and complexity are in the lower ranges to enable further modification and decoration during optimization programs. For the cell painting assay, a positive correlation between molecular weight and hit rate was observed.^36^ In addition, target-based activity correlates with spacial complexity.^81^ The Enamine compounds and the in-house PNPs may be very different in this respect, which may explain the lower hit rate observed for the selected Enamine-PNPs.

These findings indicate that a wide range of PNPs can readily be obtained commercially from Enamine, and most likely also from other vendors, and that from these PNPs, compounds with unexpected and probably new bioactivity will be identified. Thus, if desired, a sizable PNP screening library with diversity in bioactivity can be readily assembled without the need to develop multistep, complexity-generating asymmetric transformations.

Similarly, given the ready commercial availability of PNPs, the fact that pharmaceutical companies regularly source compounds from vendors, and the observation that PNPs have frequently been synthesized in drug discovery for decades, it is to be expected that the compound decks of the pharmaceutical industry will also be enriched in PNPs.

The NPFC tool can comprehensively analyze compound structure in a given database for NP fragment and PNP content, but it still can be improved. For instance, edge fusion of an aliphatic and an aromatic NP fragment is a valid new structure for a PNP. However, for cheminformatic reasons, the computational tool will classify such a fusion as NonPNP.

In the cheminformatic analysis, the two carbons of the aliphatic fragment that are fused with the aromatic fragment are recognized as part of the aromatic system and do not match the aliphatic carbons of the aliphatic system in a substructure search. From a cheminformatics perspective, it is necessary to distinguish between aliphatic and aromatic atom types in substructure matches, since otherwise aliphatic and aromatic rings would match each other and it would not be possible to distinguish between aliphatic and aromatic systems. Hence, aromatic–aliphatic edge fusions are not recognized as PNP fragment type combinations in the computational analysis by the current version of the tool. To enable the comprehensive analysis of large databases for PNP content, this limitation of fragment identification by substructure match was accepted in the development of the NPFC tool, and future versions of the software should be designed to overcome this limitation.

In particular, this misassignment of valid PNP structures as NonPNPs by the currently available NPFC tool was accepted since the classification by the NPFC tool will actually be a lower limit of PNPs in the databases analyzed, and the true number of PNPs in ChEMBL v32 and the Enamine database will be even higher than discussed here.

Still, in accordance with the PNP definition, edge fusions of aromatic and aliphatic NP fragments can correctly define the PNPs. In the case of doubt, the reasoning according to the PNP definition should be preferred, even if the tool does not recognize structures in question as PNPs.

## 6 Pseudonatural Products in Drug Discovery

While the PNP concept dates from 2018 to 2020,^13–17,22^ it has become clear that PNPs have been synthesized in drug discovery projects over several decades. Some 32% of all compounds in ChEMBL version 32 are classified as PNP (Figure 12) and a large number of PNPs are available commercially (Figure 13).^36^ The importance of PNPs to recent successful drug design is illustrated by their marked increased appearance over time in clinical compounds (phase 1–3 and marketed drugs) found in ChEMBL version 32, from <10% in the 1950s to 20% in the 1990s, rising to a remarkable 67% among those invented since 2010,^37^ more than double the PNP content of all the ChEMBL compounds.

### 6.1 Why Are Clinical Phase PNPs Increasing over Time?

As described above, PNPs require defined nonbiosynthetic connections between NP fragments, many of which are simple aromatic and aliphatic ring systems. Aromatic and aliphatic ring content in clinical compounds, normalized to ring counts per 100 heavy atoms, shows a marked increase over time in heteroaromatic rings and a decrease in carboaliphatic rings, with carboaromatic and heteroaliphatic rings changing little (Figure 14, panel a). This has resulted in 70% of post-2010 clinical compounds containing both hetero- and carboaromatic rings, the dominant class since the 1990s (Figure 14, panel b). Increased application of nitrogen-containing heterocycles is evident in approved drugs since 2013,^82^ and increases in aromatic nitrogen atom count^12^ coincide with the increased fraction of clinical PNPs (Figure 14, panel c). However, increases in PNPs lacking heteroaromatic rings are also seen in carboaromatic clinical compounds (Figure 14, panel c), although this class, which was dominant until the 1980s, markedly declined over time (Figure 14, panel b). Consistent with these observations, post-2008 clinical PNPs, when compared to clinical NonPNPs, possess on average: 1.13 more heteroaromatic rings; 1.67 more aromatic nitrogen atoms; 0.36 fewer carboaromatic rings; 0.23 more carboaliphatic rings; and 0.40 more heteroaliphatic rings.^37^ In addition, relative to NonPNPs, PNPs are on average more polar (PSA increased by 10.9 Å^2^) and more rigid (rotatable bond count decreased by 1.13). Collectively, these differences result in PNPs occupying a distinctive chemical property space in clinical compounds (Figure 15).

**Figure 14:**
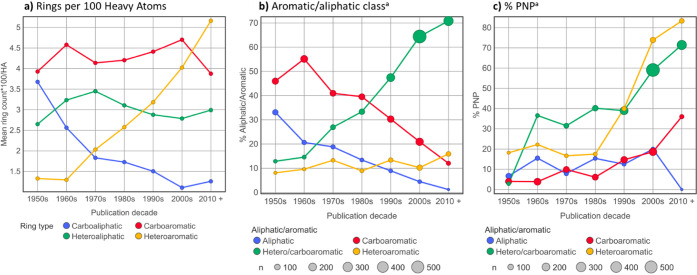
a) Ring-type density (ring count normalized to number per 100 heavy atoms), b) aromatic/aliphatic compound type, and c) % PNP in clinical compounds (phase 1–3 and marketed drugs) from the ChEMBL v32 data set^37^ over time. Publication date is the first appearance in Scifinder. ^a^Aromatic/aliphatic classes: aliphatic = 0 aromatic rings; carboaromatic = ≥1 phenyl rings and 0 heteroaromatic rings; hetero/carboaromatic = ≥1 phenyl rings and ≥1 heteroaromatic rings; heteroaromatic = ≥1 heteroaromatic rings and 0 phenyl rings.

**Figure 15:**
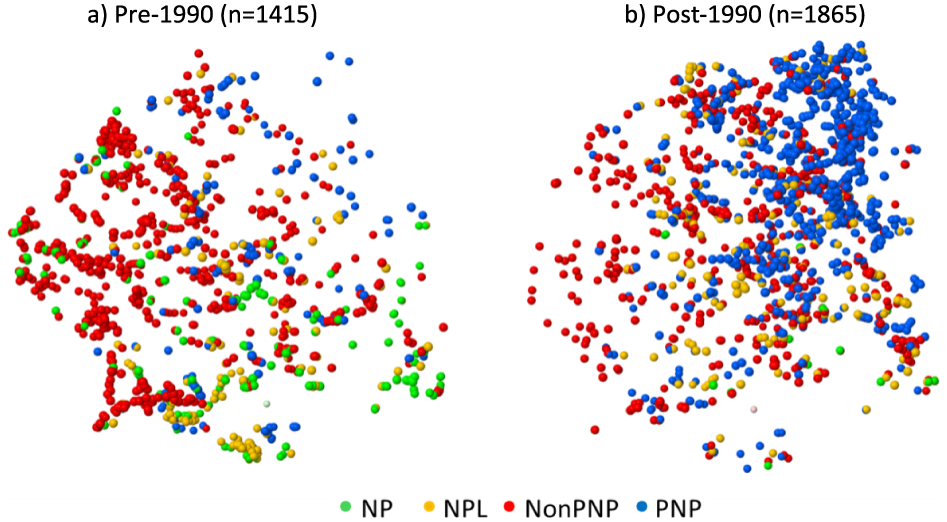
Distribution by publication period, pre- and post-1990, of PNP, NonPNP, NP, and NPL clinical compounds in property space defined by t-distributed stochastic neighbor embedding (t-SNE). 3-D coordinates derived from MW, ALogP, cx_LogD, HBA, HBD, PSA, RotB, Fsp3, stereocenters, normalized special score (nSPS), carboaliphatic rings, carboaromatic rings, heteroaliphatic rings, heteroaromatic rings, and aromatic_N atoms. t-SNE calculation was done using DataWarrior. Clinical compound publication dates are from Scifinder.^37^

Rather than being the result of deliberate “NP-based” design, i.e., the purposeful *de novo* combination of NP fragments to yield PNPs, we surmise it is likely that the application and increased recent use of PNPs has arisen because many NP fragments and ring systems, especially heteroaromatics, are dominant components in the armory of structures, building blocks, and synthetic methodology regularly used by medicinal chemists. In addition, as approved drugs and clinical compounds are increasing in size over time, the observed coincident increase in heteroaromatic over carboaromatic rings,^12^ seen in Figure 14a, helps keep lipophilicity under control, thereby reducing overall developability risks.^83^ The role of transporters in facilitating the passage of compounds across membranes to enable oral absorption or intracellular activity should also be considered in this context, given the recognition events that evolved to enable such processes.

Contemporary drug discovery is fixated in defining or expanding chemical space, potentially overinfluenced by the thinking described by Lipinski and coworkers in their rule of 5 (Ro5), especially the molecular weight limit of 500. Emerging successful features of “beyond the Ro5” drugs include limited conformational flexibility and intramolecular polarity shielding, while the increase in heteroatoms (especially hydrogen bond acceptors) accompanying molecular weight increase is implicit, being necessary to modulate lipophilicity as described above.^84,85^ The useful predictor of permeability and oral bioavailability based on log *D*_7.4_ and calculated molar refraction (CMR) developed at GSK is underscored by compelling statistics, in spite of some drugs with good oral exposure bucking the trends.^86^ That the majority of these outliers are natural products should not go unnoticed (these are in line with provisos in the original Ro5 publication), with connotations regarding their probable recognition by transporters. Mapping of transporter recognition might lead to a better understanding of the “chemical space” where natural products and NPL and PNP molecules offer keys to enable expansion into those regions that other molecules effectively cannot reach. Significantly, the predominance of NPs in antimicrobial compounds that are outliers in the log *D*_7.4_/CMR analyses was noted in this context.^12^

### 6.2 Increased Abundance of PNPs in Clinical versus Reference Compounds^37^

The appearance of PNP, NonPNP, NPL, and NP compounds (PNP_Status, see above for definitions) in clinical compounds, together with clinical target-matched reference compounds, from ChEMBL version 32, reveals that in post-2008 publications, clinical compounds are relatively more enriched in PNPs (compare Figure 16, panels a, b). This difference holds up across most target classes, with notable exceptions being the highly explored protein kinases and aminergic GPCRs (Figure 16, panel c), with ∼70% and ∼40% PNP content, respectively, in both clinical and reference compounds. Overall however, a post-2008 clinical compound is 54% more likely to be a PNP than a reference compound. In addition, two further NP metrics, namely the fraction of NP fragment heavy atoms in the compound’s Murcko scaffold, and the NP-likeness score,^87^ are similarly increased across most target classes in clinical versus reference compounds.

**Figure 16:**
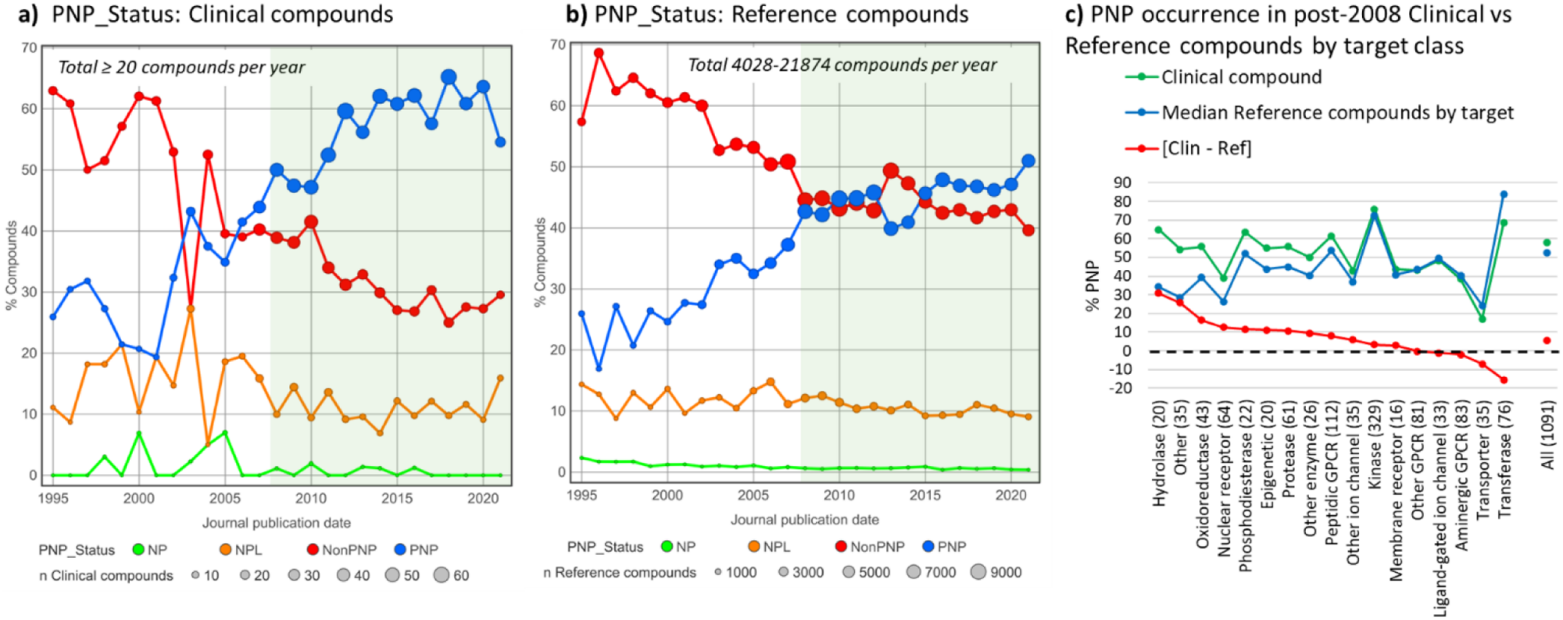
PNP_Status (defined in Section 5) vs publication date. **a)** For clinical compounds phases 1–3 and marketed drugs and **b)** reference compounds acting at the same biological targets as the clinical compounds. **c)** % PNP in clinical compounds, reference compounds by target, and the [clinical-reference] difference; target class clinical compound numbers in parentheses. Figure adapted from Heinzke et al.^37^ under CC-BY 4.0.

In 1163 post-2008 clinical compounds, 176 different NP fragments were observed, with 842 different combinations of NP fragment pairs among the PNPs. Fragment combinations in the clinical PNPs are also dominated by monopodal (69%) and fused edge (16%) connections found in the full ChEMBL set (Figure 12). The 58 most commonly used fragments (Figure 17) make up 90.5% of all fragments used. Among these 58 NP fragments, 15 occur more frequently in clinical versus reference compounds, and 4 are increased in reference compounds (Figure 17). Clinically preferred NP fragment combinations are seen in 12 of the 31 most common PNP combinations, accounting for 22% of the total clinical occurrence. Pyrrolidine and cyclopropyl rings, individually and in combination with other fragments, commonly have higher abundance in clinical over reference compounds.

**Figure 17:**
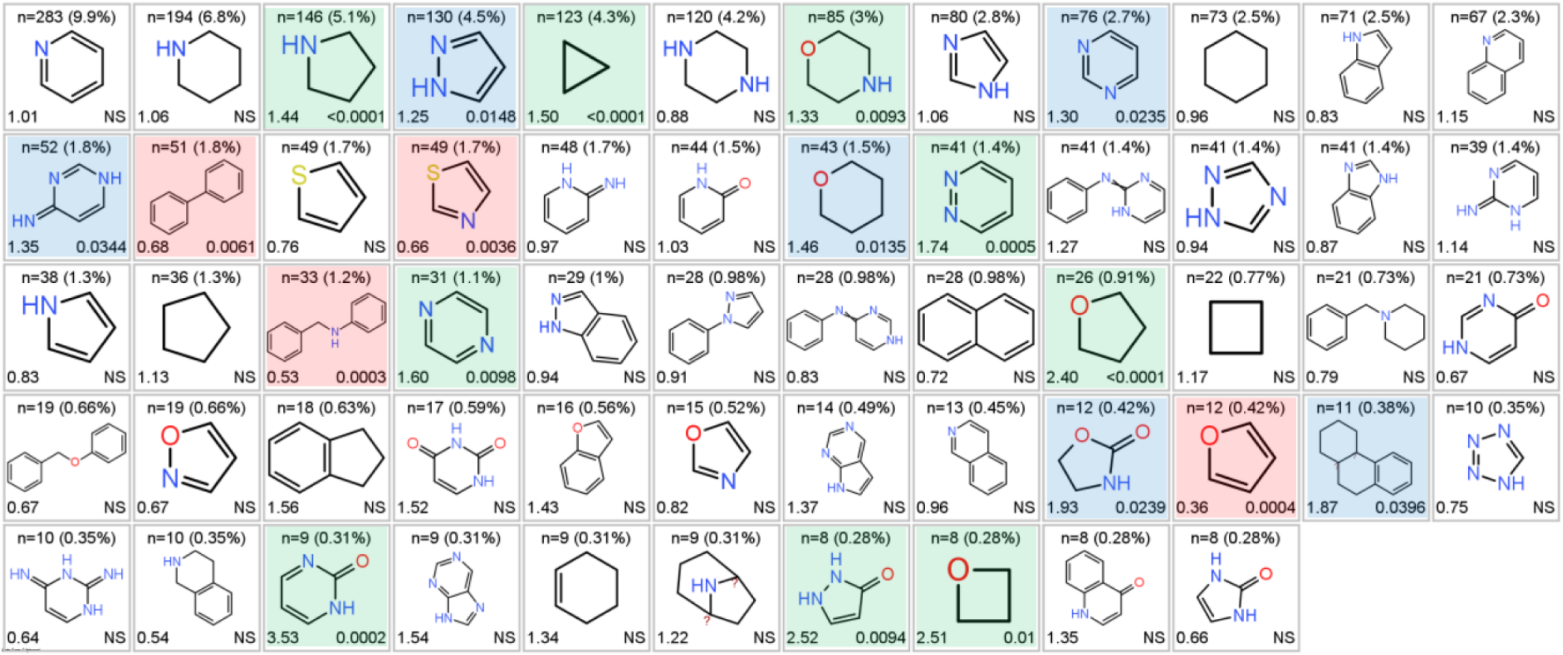
58 most abundant post-2008 clinical NP fragments comprised 90.5% of total clinical NP fragment count. Shown for each fragment are count (% clinical fragments) (top); odds ratio vs reference compounds (lower left); *p* value (lower right; NS = not significant, *p* > 0.05). Clinical abundance increased (15, 26%): green, *p* < 0.01; blue, *p* = 0.01–0.05. Clinical abundance decreased (4, 7%): red (pink, *p* < 0.05). Canonical tautomers shown, as generated by RDKit. Figure 17 adapted from Heinzke et al.^37^ under CC-BY 4.0.

Recently,^88^ it has been reported that the proportion of clinical compounds with high NP-likeness^87^ values of >0.6 increases through development phases as follows: phase 1, 19.8%; phase 2, 21.4%, phase 3, 25.6%; FDA approved 24.5%. However, NP-likeness is known to be decreasing over time in drugs.^12^ From our ChEMBL data set,^37^ the proportion of clinical compounds (phases 1–3 and marketed drugs) with NP-likeness >0.6 reduces by decade of first disclosure as follows: 1950s, 37.3%; 1970s, 25.7%; 1990s, 19.7%; 2000s, 7.5%; 2010 onward, 4.9%. The published study^88^ values are seemingly heavily influenced by pre-2000 compounds and it is likely that the phase 1 compounds used possess lower NP-likeness values than the later phases because they would have been discovered more recently. Further studies taking into account the impact of the discovery date on NP-likeness in clinical compounds are required to definitively examine any attritional changes between development phases. Nevertheless, the overall clinical versus nonclinical picture using post-2008 target-matched compounds,^37^ summarized above, indicates that a degree of “natural selection”, where NP fragments are increasingly incorporated and PNP fraction increases, may occur as compounds are optimized toward clinical candidates. This aspect is examined in the next section.

### 6.3 PNPs Increase as a Result of Optimization

Recent literature compilations of start molecule to finish molecule optimization pairs, covering hits-to-candidates published in 2018–2021 (*n* = 156)^89^ and fragments-to-leads published in 2015–2022 (*n* = 198),^90^ were examined to assess if changes in NP character occur during optimization. The overall changes in PNP_Status seen in optimization with these data sets show a 32% increase in PNPs in the hit-to-candidate set and a 134% increase in PNPs in the fragment-to-lead set (Figures 18, panels a1 and 18, panel b1, respectively). The candidate molecules are of course further optimized compared to the fragment-derived lead molecules and also possess a greater proportion of PNPs (64% versus 55%). Starting fragments show a low proportion of PNPs (23% versus 48% of the starting hits), which is to be expected because molecular weight control is a necessary prerequisite in fragment selection (typically <300 g/mol),^90^ limiting the numbers of possible constituent NP subfragments. In contrast, molecules in the hit set have higher molecular weight and the majority (59%) are already partially optimized, as they originate from the published literature.^89^

**Figure 18:**
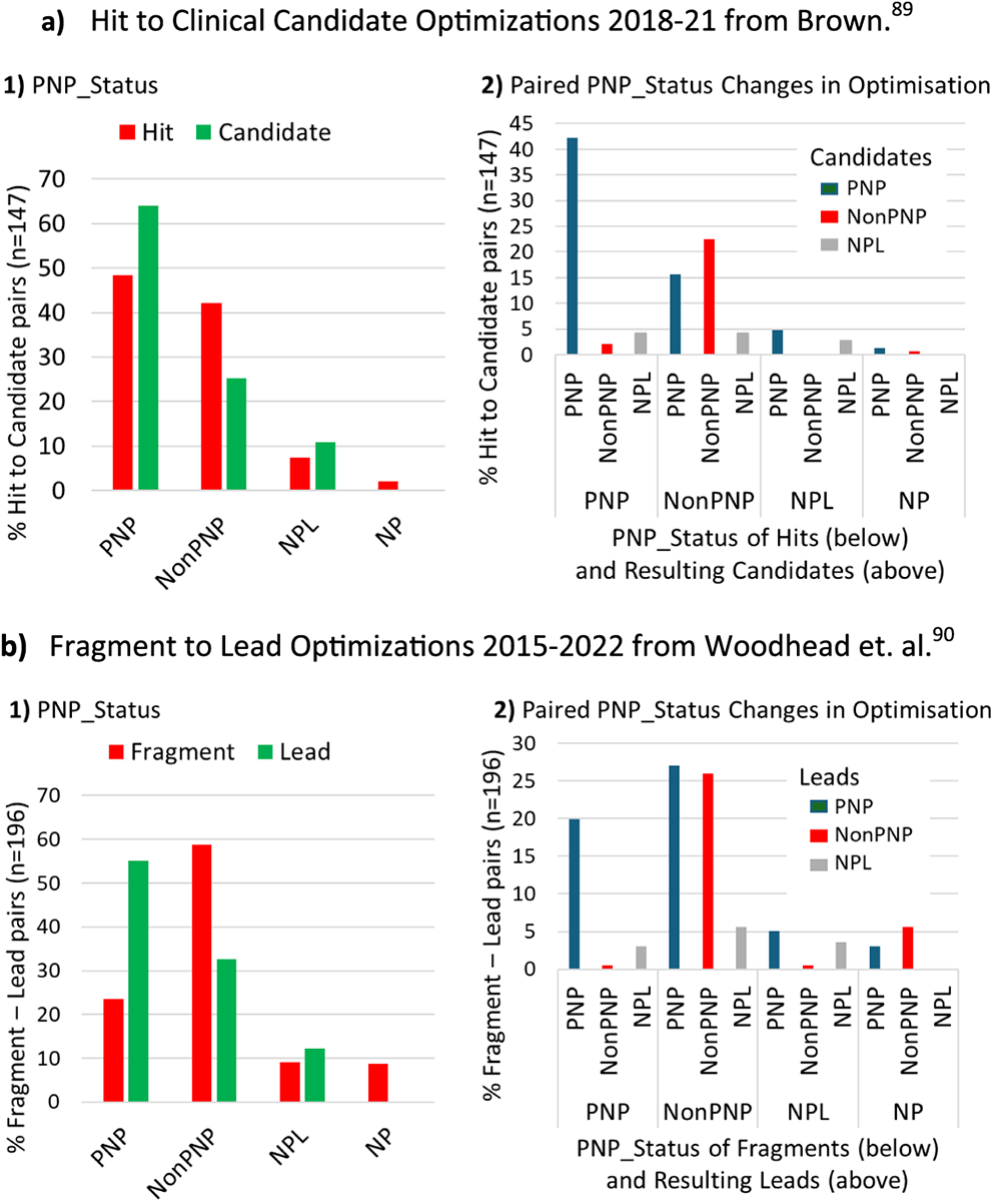
Overall changes in PNP_Status (a1, b1) and paired PNP_Status changes (a2, b2) seen in compilations of hit-to-candidate optimizations (**a**) and fragment-to-lead optimizations (b). In **a),** mean NP fragment counts are 2.05 for hits and 2.60 for candidates (*p* < 0.0001); mean NP likeness values are −0.90 for hits and −0.89 for candidates (not different). In **b)**, mean NP fragment counts are 1.25 for fragments and 2.37 for leads (*p* < 0.0001); mean NP likeness values are −0.93 for hits and −0.92 for leads (not different).

Changes in PNP_Status occurring in optimization show consistent trends in both sets (Figure 18, panels a2 and b2). Specifically, in the hit-to-candidate set, 87% of hit PNPs produce candidate PNPs and 90% of the candidate NonPNPs come from NonPNP hits; in the fragment-to-lead set, the corresponding values are 85 and 80%. Increases in NP fragment count correspondingly occur in both data sets, but there is no change in NP-likeness scores (values given in Figure 18 caption). Collectively, these observations are consistent with the large scale ChEMBL analysis,^37^ suggesting that application of NP fragments tends to increase as optimization progresses, from early lead generation through to candidate selection, resulting in an increased proportion of PNPs. In particular, starting optimization with a PNP is highly likely to provide an optimized PNP, while NonPNP starting points are about equally likely to become optimized to PNPs or NonPNPs.

It is important to note there is no comparable “non-NP” fragment set, and it is possible that the proportions of some synthetic non-NP fragments might also increase among clinical compounds as a result of optimization. However, the NonPNP to PNP optimization trend could be of potential value in both the design and selection of chemical libraries for high-throughput and focused screening.

### 6.4 Examples of NonPNPs Optimized to PNP Candidate Drugs

Moving from a NonPNP to a PNP is the dominant change in the PNP_Status observed in the optimization pairs (Figure 18). A handpicked selection of recent illustrative examples of PNP candidate drug discovery from NonPNP chemical starting points is shown in Table 2. The starting compounds in Table 2 originate from various screens (focused, high-throughput, virtual, DNA encoded library) as well as from reports in the literature. In these particular examples, it is apparent that a number of optimization tactics, coded by color in Table 2, result in the application of NP fragments and creation of PNPs, including:

**Table 2:**
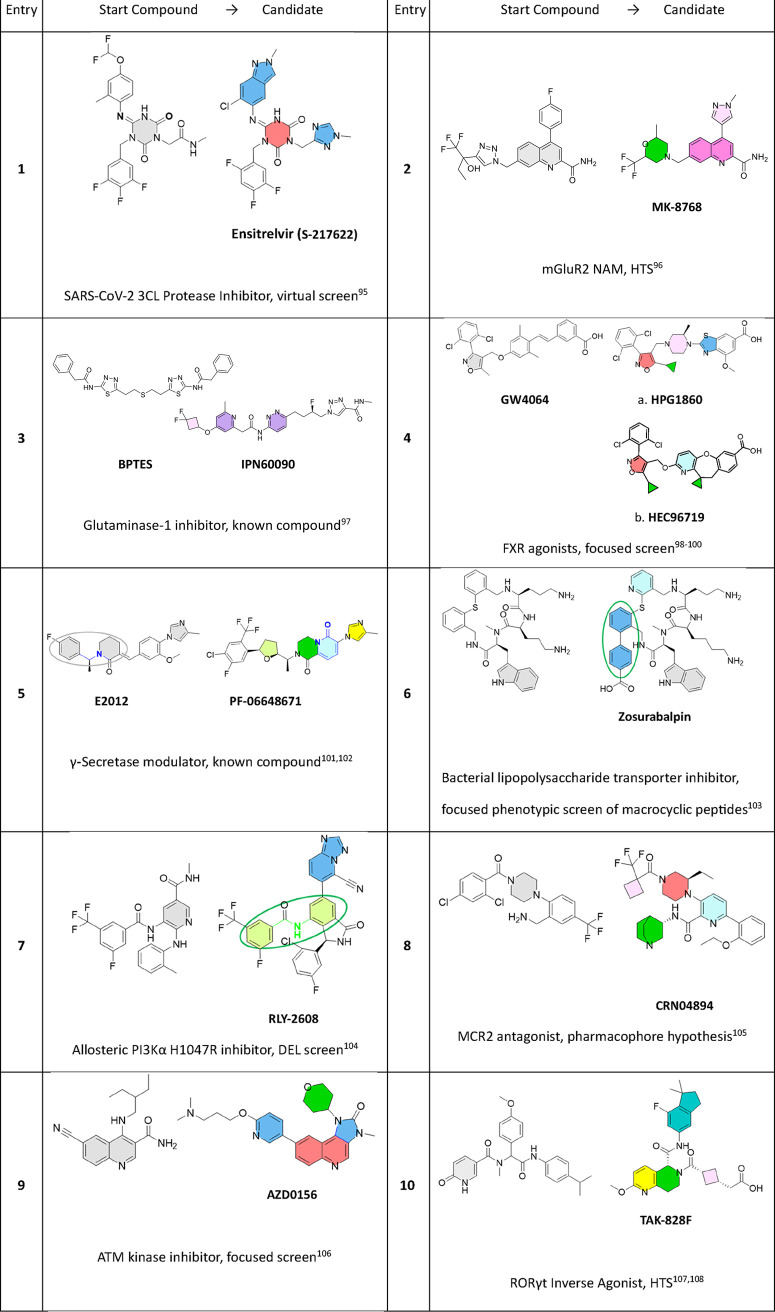

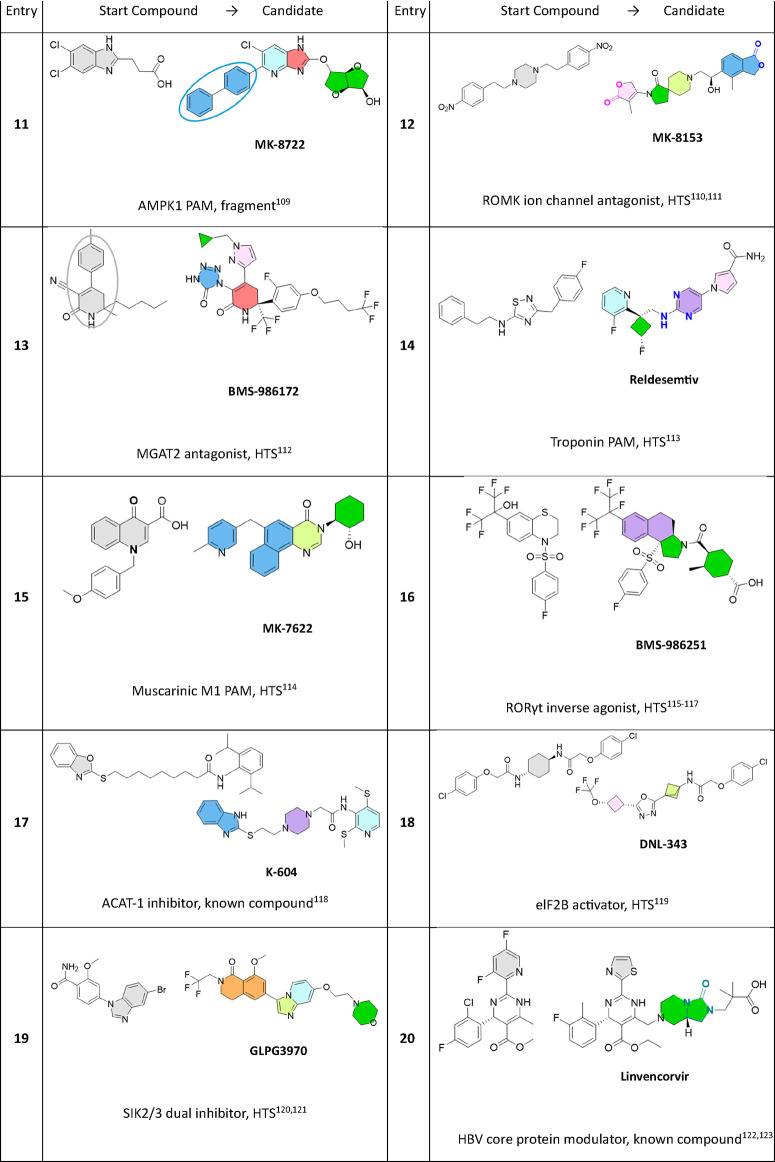
Examples of the Optimization of NonPNP Start Compounds to PNP Candidates^95–123^

a NP fragments are shaded. The NP fragments in the PNP structures are colored, according to strategies 1–8 employed (see main text for more details) as follows: 1) (red color) modifying substituents to NP fragments while retaining the starting NP core fragment; 2) (light green color) replacing the core NP fragment in the start compound with another NP fragment; 3) (purple color) introducing core NP fragments; 4) (blue color) adding aromatic NP fragments (and replacing non-NP aromatics); 5) (light blue color) NP phenyl bioisosteres: converting aromatic C or CH to aromatic N; 6) (pink color) other NP phenyl bioisosteres; 7) (green color) added aliphatic NP fragments not covered by strategies above; 8) (yellow color) other NP fragment retained from the start point; 9) (orange color) other. In entry **20**, the candidate has two added fused-ring NP fragments.

1) Modifying substituents to NP fragments while retaining the starting NP core fragment: entries **1, 2, 4a, 4b, 8, 9, 11, 13** (red).

2) Replacing the core NP fragment in the start compound with another NP fragment: entries **5, 7, 11, 12, 15, 18, 19** (light green).

3) Introducing core NP fragments: entries **3, 10, 14, 16, 17**(purple).

4) Adding hydrophobic NP fragments: entries **6, 7, 9, 10, 11, 12, 13, 15** (blue).

5) Use of NP phenyl bioisosteres. a) Converting aromatic CH to aromatic N (the “necessary” nitrogen):^91,92^ entries **4b, 5, 6, 8, 11, 14, 17, 19** (light blue).

6). Other NP phenyl bioisosteres: entries **2, 3, 4a, 8, 10, 12, 13, 14, 18** (pink).

7) Incorporation of aliphatic NP fragments is common in this set, with only three candidates (entries **1, 6,** and **7**) exclusively employing aromatic NP fragments. Aliphatic NP fragments not covered by strategies above are shown, entries **2, 4a, 4b, 5, 8, 9, 10, 11 12, 13, 14, 15, 16, 19, 20** (green).

8) Other NP fragment retained from the start point: entries **5, 10** (yellow).

9) Other: entry **19** (orange).

These tactics overlap and can be applied simultaneously. While the analysis in Figure 14 shows the importance of aromatic heterocycles in PNPs, the examples in Table 2 show that hydrophobic and aliphatic NP fragments may also be regularly applied to obtain PNPs from NonPNPs. Common aromatic heterocycles (Figure 17) are NP fragments, and a number of fused bicyclic heterocycles based on these, that can act as potential phenyl isosteres,^93^ are PNPs. The phenyl ring itself is ubiquitous in nature and for this reason was not included among the NP fragments,^79^ but there is much recent interest in the application and synthesis of aliphatic sp^3^ carbon-rich phenyl ring isosteres. The bulk of these isosteres,^94^ including bicyclo[1.1.1]­butane, bicyclo[2.1.1]­hexane, bicyclo[2.2.1]­heptane, bicyclo[3.1.1]­heptane, spiro[3.3]­heptane, bicyclo[2.2.2]­octane, stellane, cubane, cuneane and ladderane, as well their aza- and oxo-derivatives, are PNPs, as they are constructed by bridging and/or fusing small-ring carbo- and heteroaliphatic NP fragments. Overall, seeking bioisosteric replacements in lead compounds is a mainstream optimization strategy to improve potency and pharmacokinetics, which often uses NP fragments and can result in the conversion of non-PNP to PNP structures.

### 6.5 Potential for a Novel PNP Design

Only 176 NP fragments are used in >1000 clinical compounds from ChEMBL published since 2008, yet they make up, on average, 63% of the heavy atoms in the compound’s Murcko core scaffolds.^37^ This aligns with the literature observations showing that known ring systems appear more frequently in drugs than new ones,^124^ and exploiting known ring and/or scaffold combinations is a fruitful discovery strategy.^125^ As discussed in the PNP design section above, the potential for developing novel scaffolds based on applying existing NP fragments is enormous, which may obviate the need to select designs from the vast arrays of “diverse” non-NP ring systems.^126,127^ There are in total 1673 NP fragments (listed in the Supporting Information in ref. 37) and a number of them, including those lacking nitrogen atoms, or with multiple rings, or with unusual functionality, will understandably be unattractive to many medicinal chemists. It should also be noted that although a fragment may be of natural origin, it may still possess undesirable toxicity or pharmacokinetic properties. A handpicked list of potentially interesting bicyclic NP fragments that occur rarely, or were absent in the ChEMBL post-2008 compound-target literature,^37^ is shown in Figure 19.

**Figure 19:**
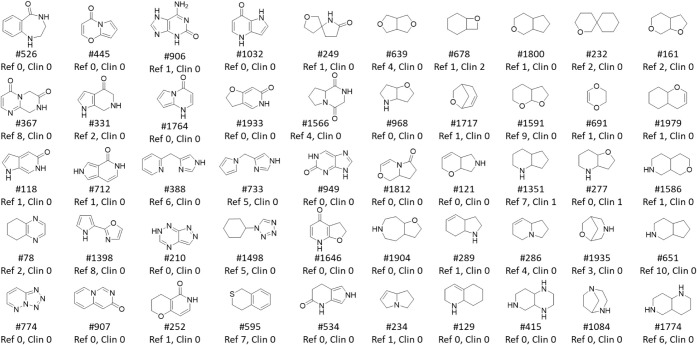
Bicyclic NP fragments that were absent or rarely seen in the 2008 onward literature,^37^ comprising clinical compounds and those reference compounds acting at the biological targets accounting for clinical efficacy. The counts of fragment appearances in reference and clinical compounds are shown. Fragment numbering is taken from the full list (available in Supporting Information in ref. 37).

Although drugs and clinical candidates are becoming less NP-like over time,^12,37^ the NP signal in biologically active compounds remains very strong because of the widespread use of NP-derived part-structures and fragments. The nonbiogenic combination of NP-derived fragments, which can be considered as ″privileged” structures, to form PNPs, despite being largely unrecognized until recently, is dominant in recent successful drug discovery. The opportunity now exists to strategically exploit the NP fragment and PNP concepts in all stages of drug design and discovery, from screening libraries to lead generation and optimization to candidate selection. For example, the databases generated describing all potential compounds containing up to 13 and 17 heavy atoms contain vast numbers of molecules which can be selected using the presence of constituent NP fragments.^128–130^

## 7 Discussion and Conclusions

Natural products and their derivatives constitute a large fraction of currently available drugs and provide ample inspiration for the discovery of new bioactive chemical matter. Several design principles have been developed aiming to capture the biological relevance and the ability to bind to multiple proteins in new natural product inspired structures.^131^ However, they may be limited in the extent of exploration of biologically relevant chemical space and biological target space.

The PNP principle aims to overcome these limitations by merging the biological relevance of NP structure with the ability of fragments to explore larger fractions of chemical space.^132^ To this end in PNP design and synthesis, NP fragments or fragment-sized NPs^20^ are linked in unprecedented combinations and arrangements to obtain scaffolds that retain the biological relevance of the guiding NPs and NP fragments, but are not accessible from current biosynthesis pathways. The combination and fusion of fragments in different connectivity patterns and in different arrangements are general strategies that can yield chemically and biologically diverse PNP collections enriched in novel bioactivity that differs from the activity of the guiding NPs.^27,34^ In particular, the combination and orientation of the fragments, in addition to the chemical structures of the individual fragments, determine the bioactivity of the PNPs, and the unprecedented NP fragment combinations may define new chemotypes for targets with or without known ligands. These insights indicate that the PNP concept enables exploration of new chemical space, thereby retaining biological relevance of the known NP structure.

The trains of thought underlying the PNP concept have inspired synthesis efforts before. Thus, Suga and coworkers described in vitro synthesized cyclic peptides embodying non-natural amino acids as “pseudo natural products”,^133,134^ and Oshima et al. intercepted biosynthetic pathways to obtain alkaloid-like compounds which were considered PNPs.^135,136^ Tietze et al.^23^ summarized reports in the literature that describe NP-like hybrid compounds which may be considered PNPs. Shavel et al. attempted to synthesize unknown alkaloids by a synthesis following the biogenetic route leading to the morphine scaffold and introduced alternative building blocks.^137,138^ Bosch et al. developed heteromorphans by replacing the benzene ring in 6,7-benzomorphans with heterocyclic rings.^139^ Synthetic biology methods, in particular precursor-directed biosynthesis, mutasynthesis, or combinatorial biosynthesis have yielded “unnatural” polyketide natural products.^140,141^ Combination of natural and non-natural structural elements gave rise to polyketide natural product analogues not accessible by biosynthetic pathways.^142^ Cheminformatics analysis of the ChEMBL database revealed that PNPs constitute 32% of the bioactive compounds listed in ChEMBL,^36,77^ and that PNPs have been synthesized and applied since decades and in a widespread manner even without a guiding principle. These findings prove that PNPs occur frequently in bioactive compounds and validate the PNP concept as an historically proven approach for the generation of new bioactive chemical matter.

By analogy, analysis of the Enamine small molecule library which is widely sourced for both chemical biology and drug discovery projects demonstrated that also this collection contains 32% PNPs.^36,80^ Thus, PNPs also define a major fraction of early phase and frequently employed research compounds that likely will give rise to the structural cores of future bioactive compounds in academia and industry.

The fact that both the historical compounds listed in the ChEMBL database and the current compounds offered by Enamine and potentially also other vendors were established before the PNP principle was introduced and applied suggests that, historically, PNPs may have mostly been designed intuitively, rather than by purposeful inclusion of NP structures. Since natural products are rich sources of drugs and bioactive compounds, most likely, structure and pattern recognition by chemists has led to widespread inclusion of these structures and their substructures in compound design for medicinal chemistry research and drug discovery (see also below).

The PNP-principle was developed as a logic that would enable capturing the information and ability of NPs to bind to biomacromolecules. This information is genetically encoded in the structures of NPs which were selected and validated in evolution as biologically relevant compound classes with privileged structures. Such natural product-inspired compound classes might overcome the problems that NPs often are not readily accessible by means of synthesis or by isolation from natural sources. Application of the PNP logic led to the design, synthesis, and biological evaluation of NP-inspired compound classes which resemble NP-structure but are different from NPs. In particular, PNPs are obtained by combination of NP-derived fragments, which follow the “relaxed” fragment definition (see above), and should preferably combine 2–4 NP fragments. This guideline is in agreement with the finding that enrichment in bioactivity is correlated with spacial complexity and molecular weight as expressed by the normalized spacial score (nSPS; bioactivity is highest for compounds with high spacial score, i.e., nSPS is between 20 and 40, and this value is correlated to MW > 400).^81^ It also mirrors the observation made for PNPs during investigation by means of the cell painting assay that there is a correlation between activity in this broadly monitoring morphological assay and molecular weight.^36^ In agreement with this conclusion is the suggestion that for PNP synthesis, chemistries will be particularly beneficial and efficient that combine reagents in complexity generating transformations, often generating one fragment and stereocenters in the course of the transformation, for instance, ring-forming transformations. Following this strategy, PNPs may be obtained with size, complexity, and richness in stereogenic centers, that resemble the often complex structures of NPs, and that are intuitively appealing to the eye of the organic and medicinal chemist as well as the chemical biologist. The PNPs developed by us, as exemplified by the examples above, mostly follow this guideline.

However, size and complexity of NPs may differ widely, and NPs themselves may be fragment-sized, i.e., they fulfill the “relaxed” fragment criteria, and a generally applicable principle for bioactive compound design needs to take this fact into account. Consequently, NP-fragments identified in the cheminformatic analysis of bioactive compounds may also be small structurally simple hetero- and carbocycles. Such small structures have frequently been employed with success in drug discovery before and without an explicit link to NP structure. This finding suggests that biological relevance and selection in evolution are at least as important for successful PNP design as structural complexity and stereogenicity. The fact that small fragments, widely used in medicinal chemistry, are related to NPs indicates that their use in PNP design is of high value and will yield new biologically relevant compound classes.

The increasing proportion of PNPs in drugs and clinical compounds, reaching 67% of those invented after 2010, clearly illustrates their current importance to drug discovery. By comparison, it is notable that fewer PNPs (47%) exist among corresponding reference compounds from ChEMBL version 32, acting at the same targets as the clinical compounds.^37^ The impact of NP fragments is indeed profound: only 58 NP fragments are used in 90% of clinical compounds reported since 2008, yet these comprise 63% of the compounds’ Murcko scaffolds.

The “enhancement” of PNPs in clinical molecules suggests that, overall, “natural selection” of NP fragments occurs in optimization processes. The analysis of two recent compilations of start-to-finish optimizations^89,90^ (Figure 18) indicates that PNPs increase mainly as a result of optimization of NonPNP starting compounds, whereas PNP starting compounds are unlikely to be modified to NonPNPs. Examples of the creation of PNPs in successful optimizations of candidate drugs (Table 2) show the effectiveness of using NP fragments as successful bioisosteric replacements.

## 8 Future Outlook

Comparison of key properties and fragments in NPs and PNPs suggests that future PNP collections should not only enrich nitrogen-rich and aromatic fragments but also include saturated oxygen-containing and aliphatic NP fragments.^79^ Combination of 2, 3, or 4 fragments is most promising, and in order to capture the broad shape distribution of NPs, different fusion patterns should be considered already in compound design such that more diverse bioactivity will result.

From a synthetic point of view, monopodal connections of two fragments will be most readily accessible by means of well-established and proven transformations. But the limited size of the resulting PNPs may also be limiting for bioactivity,^36^ such that in the interest of chemical and biological diversity and bioactivity they should not be enriched too much. Rather, PNP synthesis should frequently employ complexity-generating stereoselective and asymmetric transformations in which, for instance, two fragments are combined with generation of a further fragment and additional stereogenic centers. Such syntheses will yield scaffolds enriched in sp^3^ centers and stereocenters and will be endowed with biological relevance and high chemical complexity and information density.^81^ Synthesis design should also avoid inclusion of dominant fragments, if possible.^34^

Numerous PNPs are commercially available as demonstrated above for the Enamine compound collection and as is most likely true for the compounds offered by different vendors. In addition, it is expected that PNPs will be widespread in corporate and academic screening collections. Thus, it is to be expected that focused PNP collections can be readily assembled if desired. In this context, compound selection should ensure that PNPs are chosen with a diverse distribution of ring and fragment number, matching molecular weight and stereochemical content. The number of compounds with the previously dominating monopodal fragment connectivity should be limited, and it should be assured that appropriate fractions of future PNP collections will represent PNPs with edge-, bridge-, and spiro fragment fusion and bipodal edge fragment connections, because they have proven to yield bioactive compound classes before.

The PNP concept builds on the evolutionary selection and prevalidation of NPs and their fragments as biologically relevant starting points in a vast chemical space. However, biologically relevant chemical space extends far beyond the structures of NPs, NP derivatives, their fragments, and combinations thereof. There are large numbers of man-made bioactive compounds and drugs that do not contain any NP structure or combinations thereof and that are composed of purely synthetic fragments, and therefore, these fragments are also biologically relevant. The PNP concept should be regarded as one of multiple possible approaches to designing and developing biologically relevant small molecules. It distinguishes itself by the fact that, by definition, it guarantees and is based on proven relevance in evolution. In a broader sense, the evolutionary algorithm that underlies the PNP concept may be applied to and include not only NP-fragments, but fragments of all known biologically relevant compounds. Extension of the concept beyond NP-structure will expand exploration of the evolutionary relevant, NP-inspired chemical space to a much wider, more general biologically relevant chemical space.

## ∇

Fraunhofer ITMP ScreeningPort, Schnackenburgallee 114, Hamburg 22525, Germany

## ○

LifeArc, Accelerator Building, Open Innovation Campus, Stevenage SG1 2FX, U.K.

## Abbreviations

Alpl: alkaline phosphatase
BIOS: biology-oriented synthesis
BD: bromo domain
C: chroman
CETSA: cellular thermal shift assay
cbe: bridge edge connection
cm: connection monopodal
CMR: calculated molar fraction
CP: cell painting
CPA: cell painting assay
CtD: complexity to diversity
DABCO: 2,6-diazabicyclo-[2.2.2]­octanes
DEL: DNA-encoded library
DOS: diversity-oriented synthesis
DNP: dictionary of natural products
dPNP: diverse pseudonatural product
ER: endoplasmic reticulum
fb: fusion bridge
fe: fusion edge
fs: fusion spiro
Gli1: glioma-associated oncogene 1
GF: griseofulvin
IDO1: indoleamine 2,3-dioxygenase 1
I: indole
MBP: median biosimilarity percentage
NP: natural product
NPL: natural product like
NPFC: NP fragment combination
nSPS: normalized spacial score
PCA: principal component analysis
PNP: pseudonatural product
PTCH1: patched 1
PQ: pyrroquinoline
QD: quinidine
QN: quinine
RHOGDI1: RHO-GDP-dissociation inhibitor 1
Ro5: rule of five
Shh: sonic Hedgehog
SM: sinomenine
SMO: smoothened
SM-O: sinomenine opened
TFIID: basal transcription factor IID
THP: tetrahydropyran
