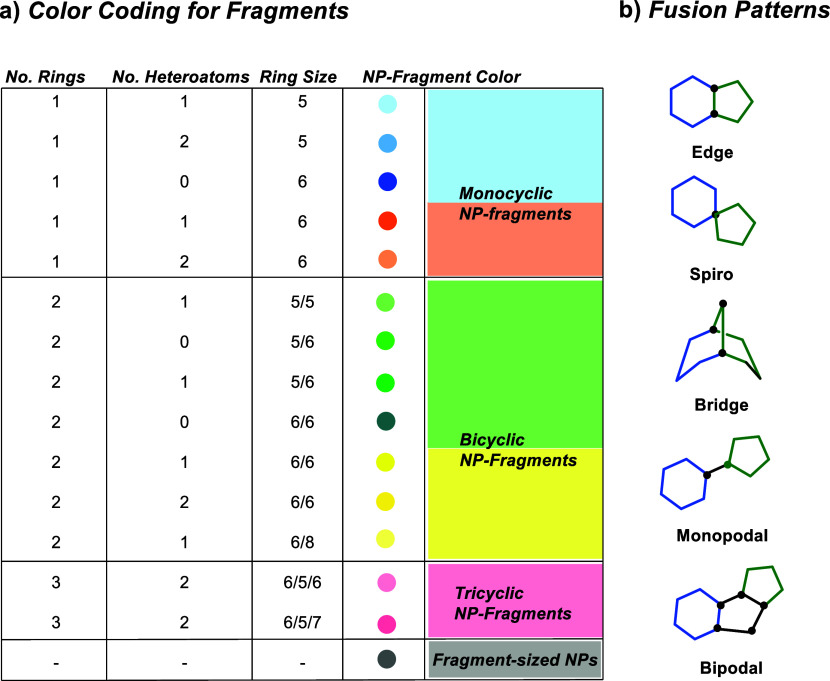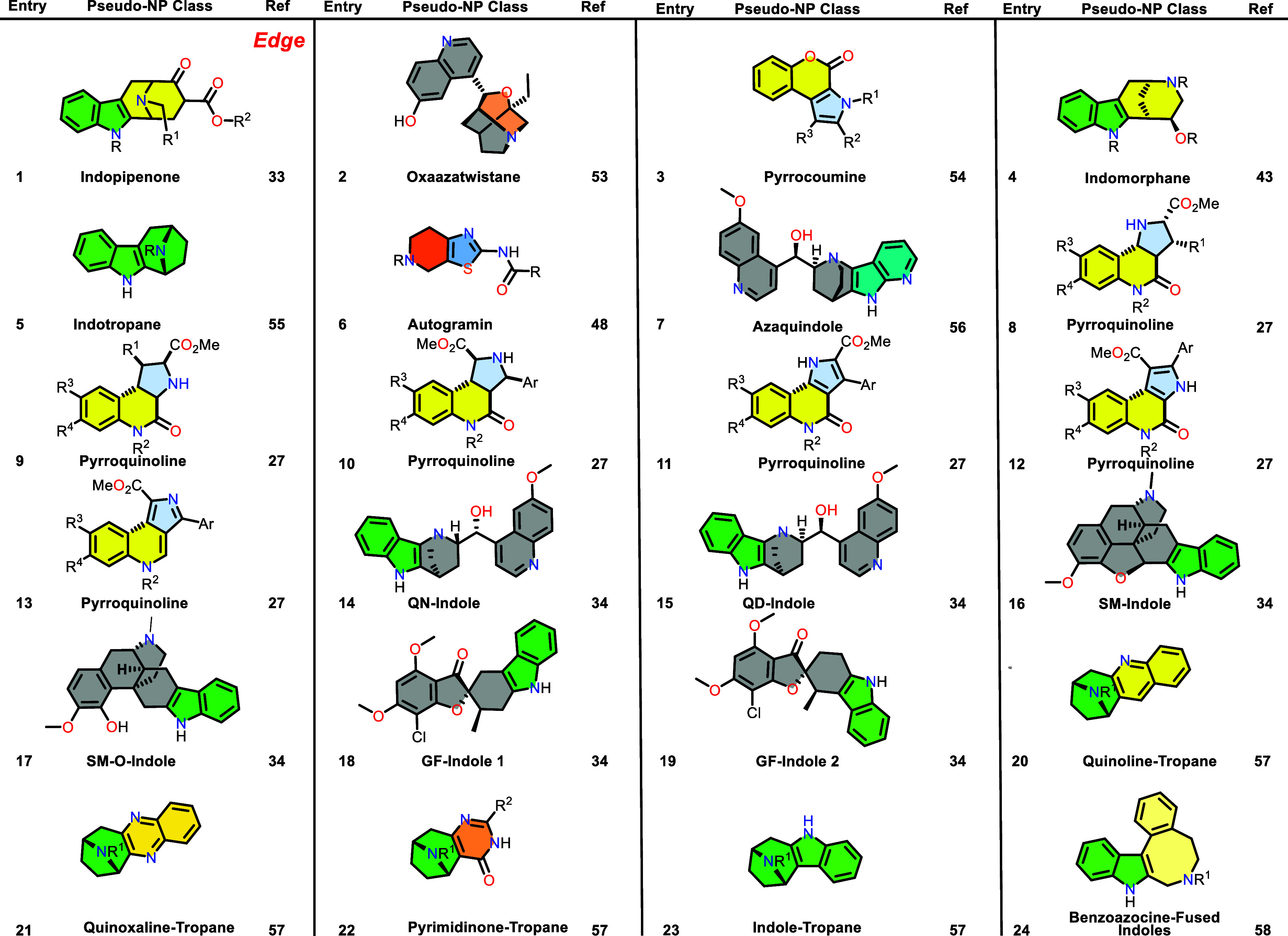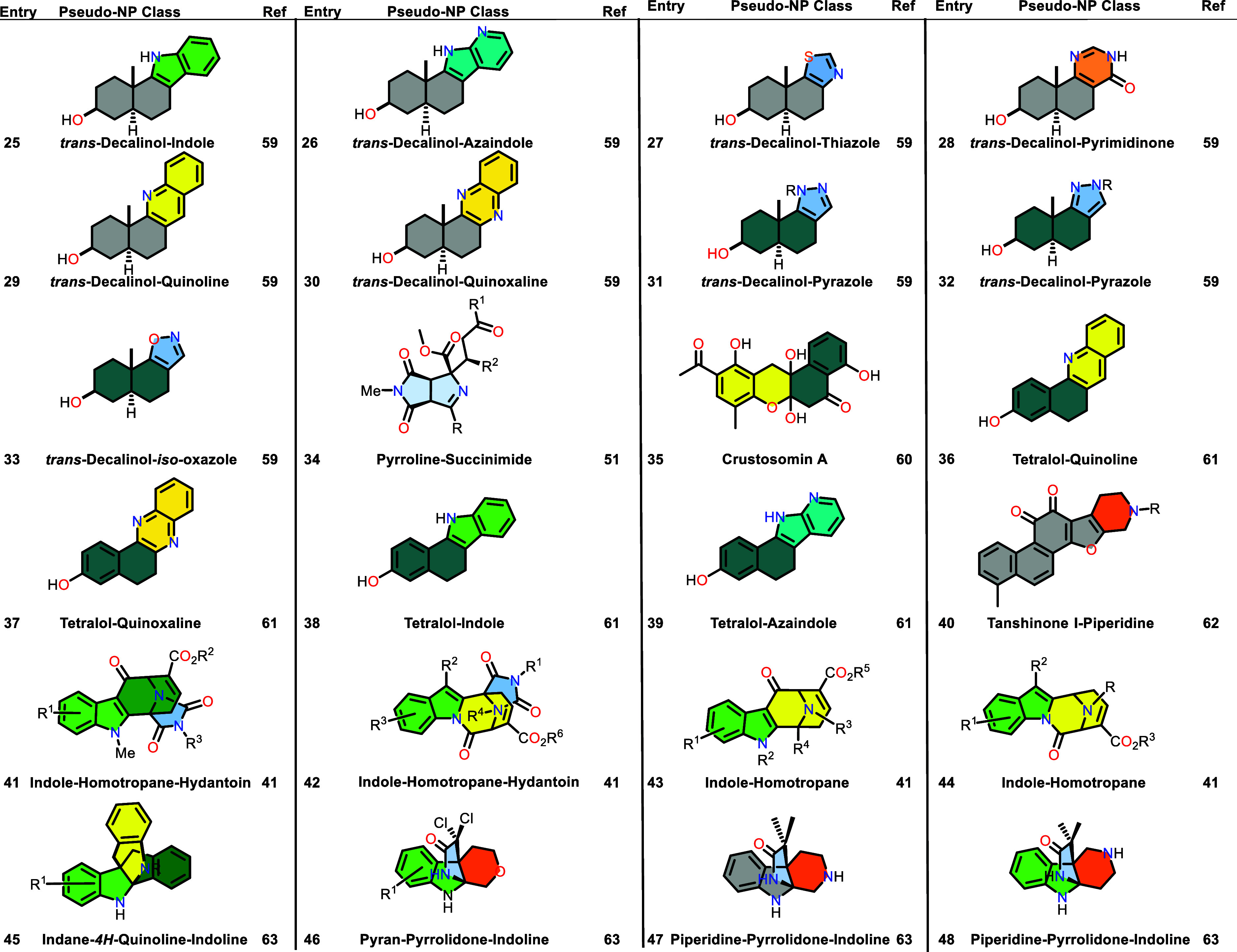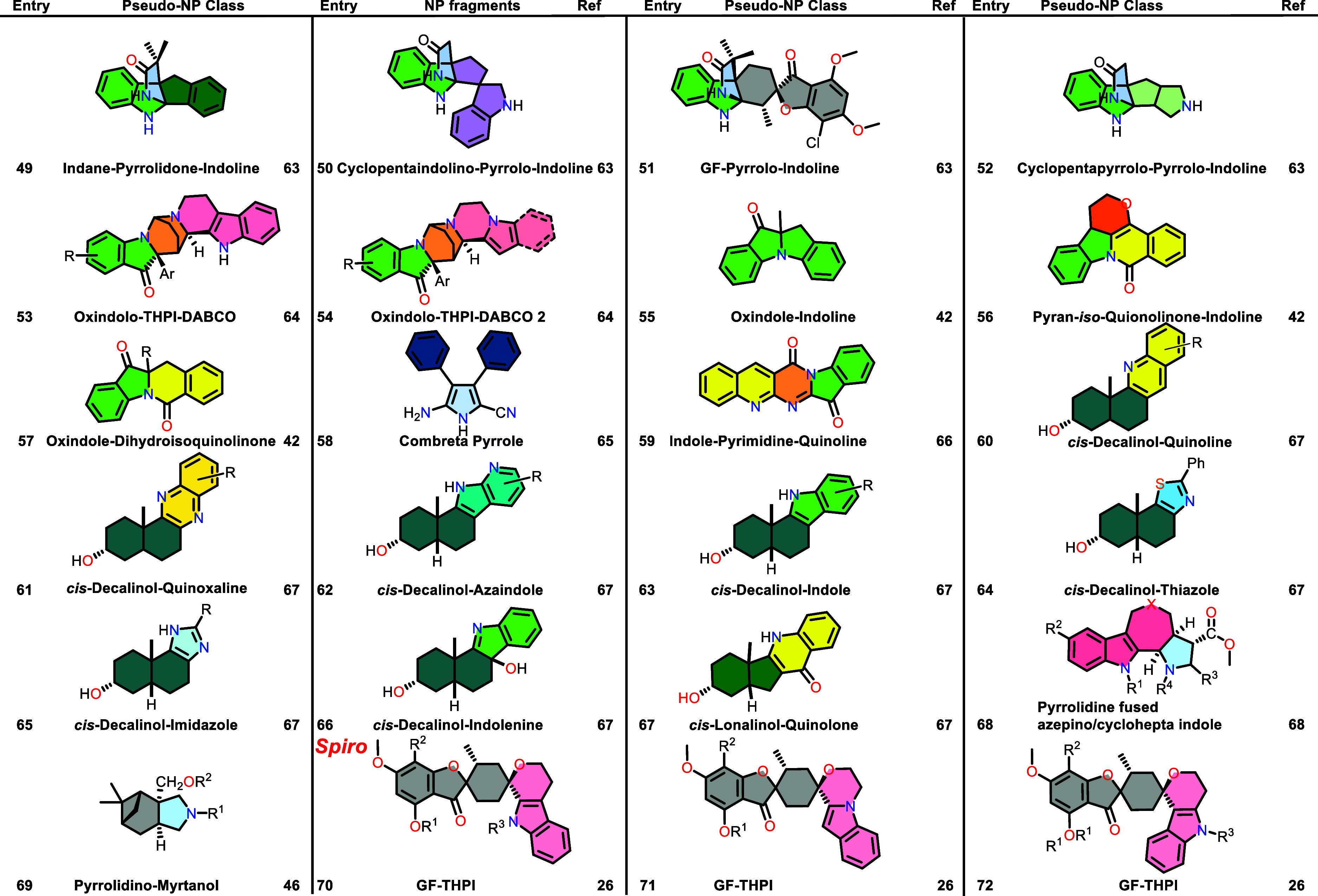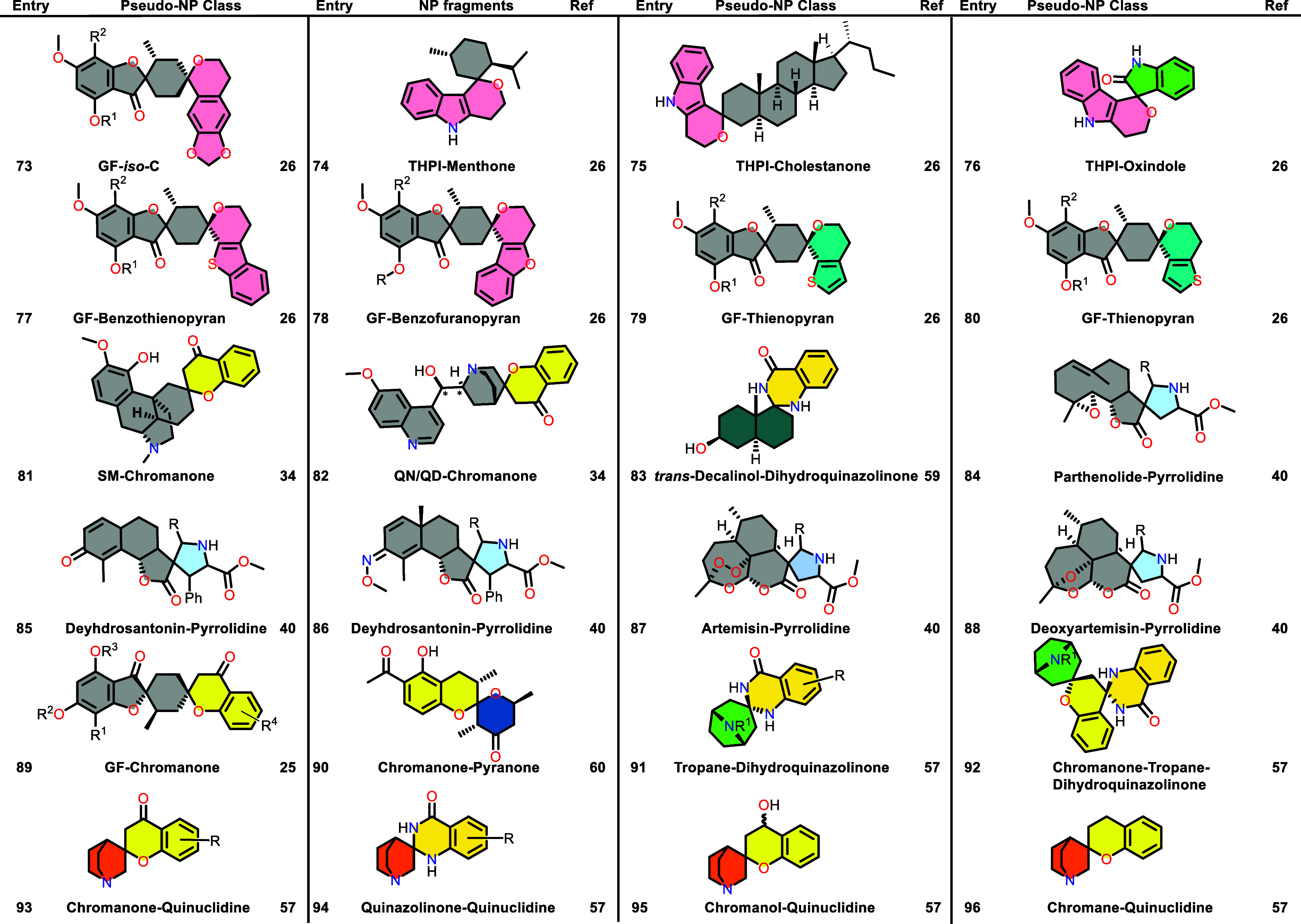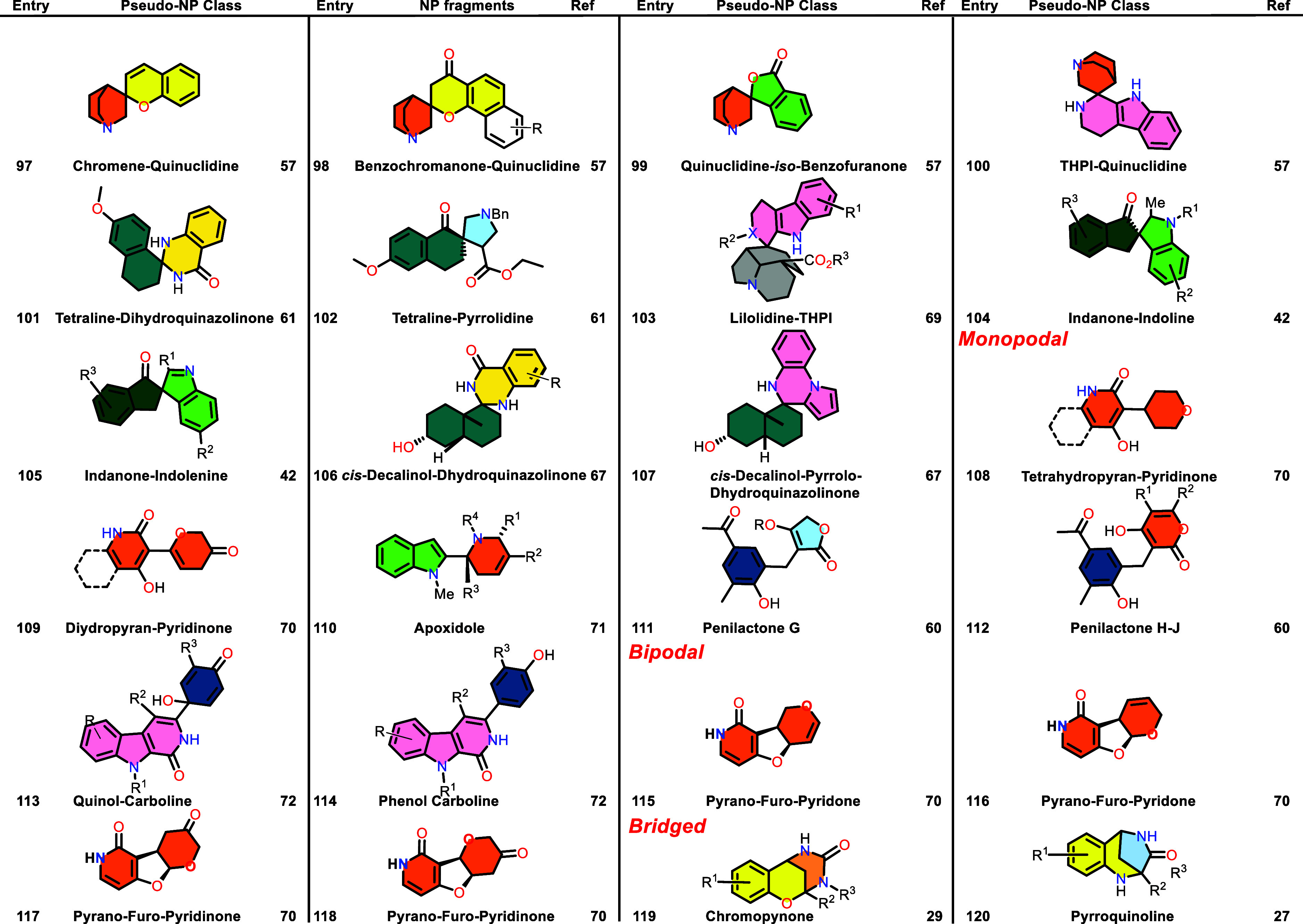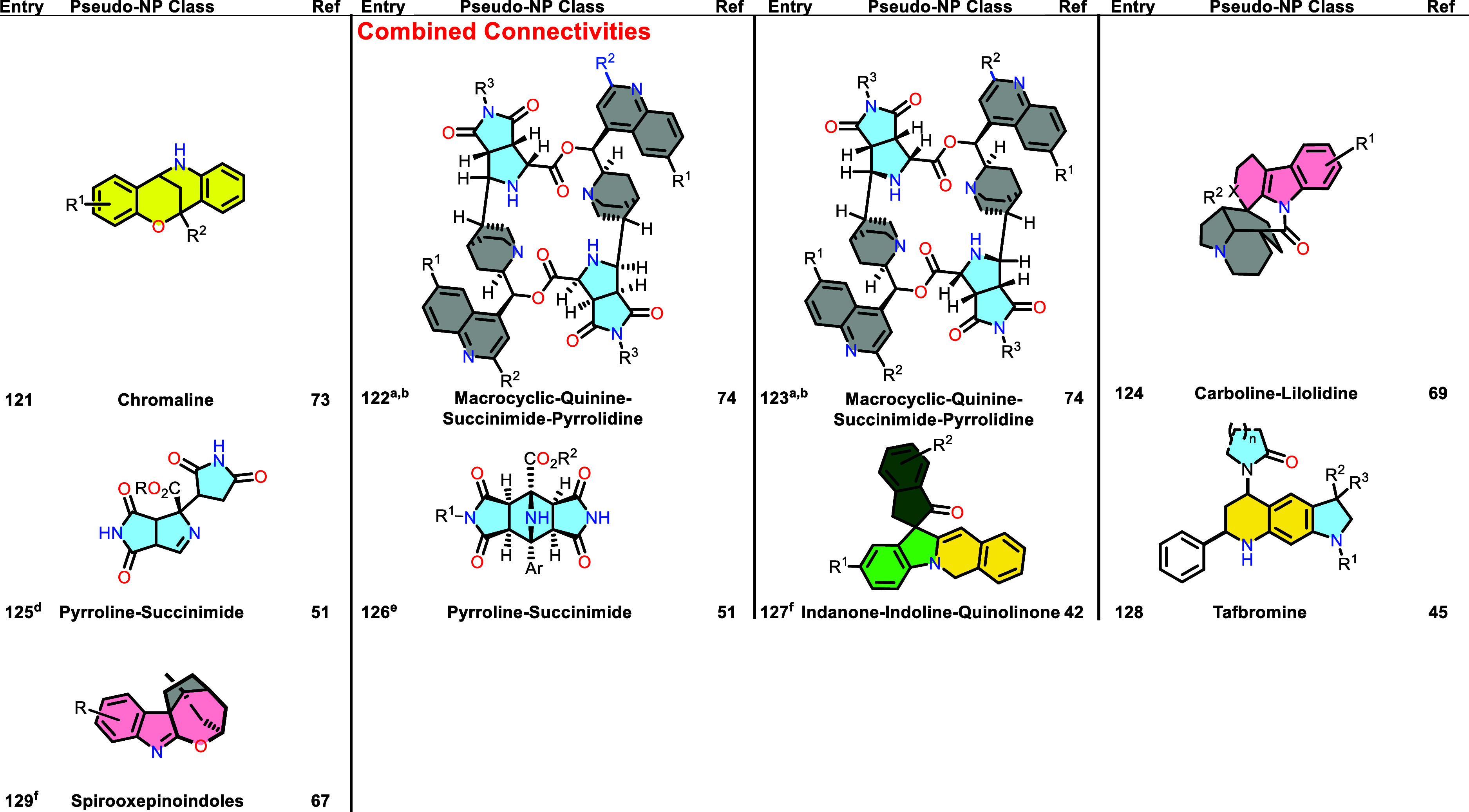# Correction to “Pseudonatural Products for Chemical Biology and Drug Discovery”

**DOI:** 10.1021/acs.jmedchem.5c02912

**Published:** 2025-10-21

**Authors:** Luca C. Greiner, Axel Pahl, A. Lina Heinzke, Barbara Zdrazil, Andrew R. Leach, Robert J. Young, Paul D. Leeson, Herbert Waldmann

Table 1 was corrected. The previous Table 1 was incomplete and a new complete Table 1 is now provided.